# Blood culture versus antibiotic use for neonatal inpatients in 61 hospitals implementing with the NEST360 Alliance in Kenya, Malawi, Nigeria, and Tanzania: a cross-sectional study

**DOI:** 10.1186/s12887-023-04343-0

**Published:** 2023-11-15

**Authors:** Sarah Murless-Collins, Kondwani Kawaza, Nahya Salim, Elizabeth M. Molyneux, Msandeni Chiume, Jalemba Aluvaala, William M. Macharia, Veronica Chinyere Ezeaka, Opeyemi Odedere, Donat Shamba, Robert Tillya, Rebecca E. Penzias, Beatrice Nkolika Ezenwa, Eric O. Ohuma, James H. Cross, Joy E. Lawn, the NEST360 Infection Group, Helen Bokea, Christine Bohne, Mary Waiyego, Grace Irimu, Ifeanyichukwu Anthony Ogueji, Georgia Jenkins, Olukemi O. Tongo, Iretiola Fajolu, Nike Olutekunbi, Cate Paul, Jitihada Baraka, Rebecca Kirby, Kara Palamountain

**Affiliations:** 1 Maternal, Adolescent, Reproductive, & Child Health (MARCH) Centre, London School of Hygiene & Tropical Medicine, London, UK; 2 Department of Paediatrics, Kamuzu University of Health Sciences, Blantyre, Malawi; 3 Department of Paediatrics and Child Health, Muhimbili University of Health and Allied Sciences, Dar Es Salaam, Tanzania; 4 KEMRI-Wellcome Trust Research Programme, Nairobi, Kenya; 5 Department of Paediatrics, University of Nairobi, Nairobi, Kenya; 6 Department of Paediatrics, Aga Khan University, Nairobi, Kenya; 7 Department of Paediatrics, College of Medicine, University of Lagos, Lagos, Nigeria; 8 Rice360 Institute for Global Health Technologies, Rice University, Texas, USA; 9 Department of Health Systems, Impact Evaluation and Policy, Ifakara Health Institute, Dar Es Salaam, Tanzania

**Keywords:** Newborn, Neonatal, Blood culture, Antibiotics, Low- and middle-income countries, Inpatient care, Antimicrobial resistance, Small and sick newborn care, Quality of care, Infection, Sepsis

## Abstract

**Background:**

Thirty million small and sick newborns worldwide require inpatient care each year. Many receive antibiotics for clinically diagnosed infections without blood cultures, the current ‘gold standard’ for neonatal infection detection. Low neonatal blood culture use hampers appropriate antibiotic use, fuelling antimicrobial resistance (AMR) which threatens newborn survival. This study analysed the gap between blood culture use and antibiotic prescribing in hospitals implementing with Newborn Essential Solutions and Technologies (NEST360) in Kenya, Malawi, Nigeria, and Tanzania.

**Methods:**

Inpatient data from every newborn admission record (July 2019–August 2022) were included to describe hospital-level blood culture use and antibiotic prescription. Health Facility Assessment data informed performance categorisation of hospitals into four tiers: (*Tier 1*) no laboratory, (*Tier 2*) laboratory but no microbiology, (*Tier 3*) neonatal blood culture use < 50% of newborns receiving antibiotics, and (*Tier 4*) neonatal blood culture use > 50%.

**Results:**

A total of 144,146 newborn records from 61 hospitals were analysed. Mean hospital antibiotic prescription was 70% (range = 25–100%), with 6% mean blood culture use (range = 0–56%). Of the 10,575 blood cultures performed, only 24% (95%CI 23–25) had results, with 10% (10–11) positivity. Overall, 40% (24/61) of hospitals performed no blood cultures for newborns. No hospitals were categorised as *Tier 1* because all had laboratories. Of *Tier 2* hospitals, 87% (20/23) were District hospitals. Most hospitals could do blood cultures (38/61), yet the majority were categorised as *Tier 3* (36/61). Only two hospitals performed > 50% blood cultures for newborns on antibiotics (*Tier 4*).

**Conclusions:**

The two *Tier 4* hospitals, with higher use of blood cultures for newborns, underline potential for higher blood culture coverage in other similar hospitals. Understanding why these hospitals are positive outliers requires more research into local barriers and enablers to performing blood cultures. *Tier 3* facilities are missing opportunities for infection detection, and quality improvement strategies in neonatal units could increase coverage rapidly. *Tier 2* facilities could close coverage gaps, but further laboratory strengthening is required. Closing this culture gap is doable and a priority for advancing locally-driven antibiotic stewardship programmes, preventing AMR, and reducing infection-related newborn deaths.

**Supplementary Information:**

The online version contains supplementary material available at 10.1186/s12887-023-04343-0.

## Key findings

**Table:** 

**1. WHAT WAS KNOWN?**
• Neonates in hospitals are at risk of healthcare-associated infections (HCAIs), many of which are antimicrobial resistant and threaten their survival • In low and middle-income countries (LMIC), HCAIs & antimicrobial resistance (AMR) are largely undetected during routine care due to limited access, or underuse, of blood culture – the current ‘gold standard’ for infection detection despite its limitations • Multi-country reviews of neonatal blood culture use are few, especially for LMIC. The NEST360 Alliance aims to report findings and learnings from implementing and scaling a small and sick newborn care package in hospitals in Kenya, Malawi, Nigeria, and Tanzania, including an assessment of neonatal infection detection
**2. WHAT WAS DONE THAT IS NEW?**
• This cross-sectional study described the gap between antibiotic prescribing and blood culture use for over 140,000 neonatal inpatients in 61 hospitals, using individually-linked clinical and microbiological data collected from newborn admission records • Health Facility Assessment data were used to classify hospitals into four performance *Tiers* based on the availability of laboratories, microbiology services, and neonatal blood culture use for those newborns prescribed antibiotics
**3. WHAT WAS FOUND?**
• We found a major gap between antibiotic prescribing (70%) and blood culture use (6%) for admitted newborns • Of the 61 included hospitals, 34 were District, 22 were Secondary/Regional/Zonal, and 9 were Tertiary/National hospitals. All hospitals had laboratories, but 24 (39%) did no blood cultures for newborns, 16 of which were District hospitals with no microbiology capacity • Most hospitals (36/61) were classified as *Tier 3* sites with microbiology services but < 50% blood culture use for newborns on antibiotics, revealing that culture is underused even when available. Importantly, two facilities had > 50% blood culture use (*Tier 4*), highlighting the potential to perform more blood cultures for hospitalised newborns in LMIC
**4. WHAT NEXT?**
• The infection detection gap for neonatal inpatients needs to be closed. Short-term, quality improvement strategies could improve blood culture use, especially at *Tier 3* hospitals missing opportunities for infection detection. Establishing active microbiology services in District hospitals remains a priority as countries aim to achieve global neonatal mortality targets • High-performing hospitals *(Tier 4)* show that doing more routine blood cultures for newborns is possible in these contexts. However, qualitative research into local barriers and enablers is required, including exploring the interface between neonatal unit and laboratory departments and how it might be strengthened • Improved blood culture use, alongside innovation for neonatal sepsis diagnostics, can enhance HCAI and AMR detection, surveillance, and outbreak control, facilitate antimicrobial stewardship programmes and reduce neonatal deaths

## Background

An estimated 30 million small and sick newborns worldwide require hospital admission each year [1]. Many receive lifesaving care but are concurrently at risk of healthcare-associated infections (HCAIs). Of the 2.3 million annual neonatal deaths [2], approximately 24% are due to infection [3], of which as many as half are attributable to HCAIs [4]. Newborns born prematurely or of low birthweight are especially vulnerable, given their immature immune systems and need for invasive interventions [3, 5]. In low- and middle-income countries (LMIC), HCAI incidence in neonatal care units is approximately nine times higher than in high-income settings due to overcrowding, resource constraints, and infection prevention challenges [6].

Less than a decade remains for countries to achieve Sustainable Development Goal 3.2 (< 12 deaths per 1000 live births by 2030). Sub-Saharan Africa has the highest number of countries requiring significant shifts in their mortality reduction to achieve this target [7]. Antimicrobial resistance (AMR) threatens progress [8, 9]. In 2019, 140,000 neonatal deaths were directly attributable to antimicrobial-resistant infections, most acquired in healthcare facilities [10]. The burden is highest in sub-Saharan Africa [10], where institutionalised births have increased over the past decade with minimal investment in inpatient level-2 newborn care [11, 12]. The *Every Newborn* Action Plan (ENAP) coverage target 4 requires 80% of subnational districts to have at least one level-2 inpatient unit to care for small and sick newborns by 2025 [13]. Care at this level includes prompt detection of neonatal infection and management with injectable antibiotics and supportive care [1, 13].

**Fig. 1:**
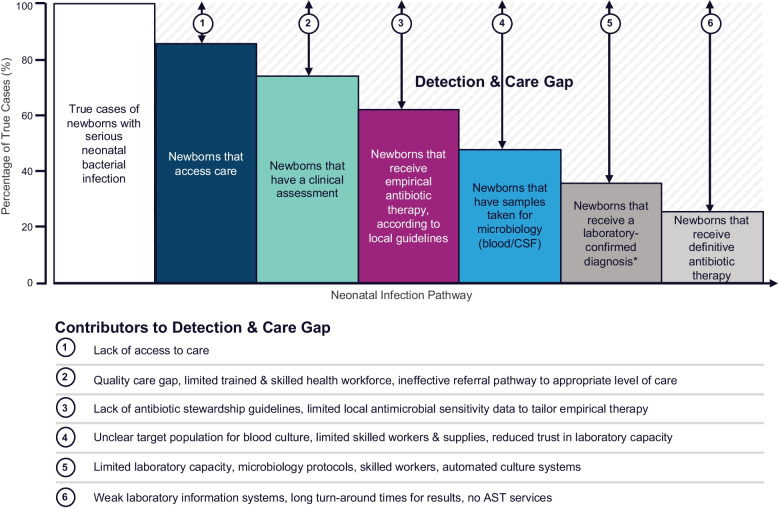
Framework for neonatal infection pathway, reflecting detection and care gaps. This paper focuses on inpatient care for neonatal infection and, therefore, does not address the first gap which arises due to lack of access to healthcare. Adapted from Lawn et al., 2017 [20] and Rahman et al., 2020 [19]. * Includes aetiology ± antimicrobial sensitivity. Abbreviations: CSF; cerebrospinal fluid, AST; antimicrobial sensitivity testing

Gaps in neonatal infection detection prevail for several reasons (Fig. 1), including limited access to, and underutilisation of, laboratory investigations. Blood culture is considered the ‘gold standard’ for detecting bacterial infections in newborns, despite drawbacks including low sensitivity and long turnaround times [14, 15]. The WHO regards blood cultures requested before antibiotic administration as a key output indicator for quality neonatal infection care [14, 16]. However, neonatal blood cultures are markedly underutilised in LMIC compared to antibiotics. For example, a published audit of almost 5,000 neonatal inpatients in The Gambia found that 4,700 received antibiotics, but only 26 had a blood culture [17]. Comparatively, in a study conducted in the Netherlands, 186 of 1024 admitted newborns received antibiotics, of whom 98% had a blood culture [18]. More recently, a multi-site study in five LMIC hospitals found that one hospital had a much higher rate of performing cultures (82%), suggesting that doing more neonatal blood cultures in less-resourced settings is possible [19].

Additional multi-country reviews of routine neonatal blood culture use, especially for LMIC, are needed to analyse the quality of neonatal infection detection in hospitals. Routine microbiological testing and data are fundamental for improving the quality of neonatal infection care, targeting equity gaps, and enabling data-driven decision-making at the individual and national levels. The dearth of routinely collected HCAI and AMR data for LMIC impedes robust surveillance systems and effective antibiotic stewardship programmes, objectives of the WHO global action plan for AMR [21].

### Objectives

This paper is part of a supplement reporting findings and learnings from NEST360, an alliance of partners, including four African governments (Kenya, Malawi, Nigeria, and Tanzania), working to reduce neonatal inpatient deaths by improving level-2 newborn care in hospitals through device installation, training, quality improvement, and HCAI detection. The aim was to quantify the neonatal infection detection gap in hospitals implementing with NEST360, addressing the following objectives:

1. Describe the gap between antibiotic prescribing and neonatal blood culture use at the hospital level.
2. Categorise hospitals into performance tiers based on laboratory and microbiology service availability (including infrastructure and human resources) and use.

## Methods

### Study setting

NEST360 produced a health systems package, including a bundle of innovative devices and data tools, implemented in 69 neonatal units across 65 hospitals in Kenya, Malawi, Nigeria, and Tanzania (some hospitals had geographically separated inborn and outborn units). This study relied on data from these hospital settings. Selected newborn statistics demonstrating differences in SSNC requirements between countries were provided in Additional file 1 (using national data from external sources). More detailed information regarding NEST360 and associated data tool development was published separately [22, 23].

### Study design and data sources

We used a cross-sectional observational study design. A Neonatal Inpatient Dataset (NID), abstracted from routine hospital records, provided individually-linked clinical and microbiological data [22]. Data collection during the study period (July 2019–August 2022) varied by hospital from 15 to 35 months, depending on timing of the health systems package installation (including equipment and training). Health Facility Assessments (HFA) were conducted at baseline (i.e., pre-installation) and involved structured, interviewer-led questionnaires with hospital staff [23]. Responses were verified by observation where possible. This study used HFA data on blood culture service availability and neonatal unit and laboratory readiness to perform cultures. Study reporting followed the Strengthening the Reporting of Observational Studies in Epidemiology (STROBE) checklist (Additional file 2) [24].

### Eligibility criteria

All 69 neonatal units across the 65 hospitals were eligible for inclusion. Hospitals were excluded if neonatal inpatient data collection had not commenced within the study period. All records for newborns aged < 28 days at admission (i.e., day 0 to 27.99 from birth), with blood culture and antibiotic data, were eligible for inclusion [25]. If newborn records were for patients > 28 days at admission, or if they lacked antibiotic or blood culture data, they were excluded from analyses.

### Data analyses

#### Neonatal inpatient data

Cleaned data were recoded (Additional file 3) and exported for analysis (Stata 17, StataCorp LLC, Texas, USA). Background characteristics of study newborns were reported (Additional file 4). All calculations were completed separately for each participating hospital, and exact 95% confidence intervals (CIs) were determined using binomial distribution. Country estimates were obtained by pooling hospital estimates and calculating the mean. Having an antibiotic prescribed was considered a proxy for a clinical diagnosis or risk of infection (i.e., the population recommended a blood culture before antibiotic administration) [16]. Blood culture use of 100% (for those newborns prescribed antibiotics) was considered the coverage target, representing current best practice guidelines [14–16]. A hospital’s neonatal unit was defined as a positive outlier if blood culture use for those newborns prescribed antibiotics was greater than the overall pooled hospital mean. Since countries have variable contexts, socio-political histories, economic policies, and newborn health indicators, no formal statistical comparisons between countries were made (Additional file 1).

#### Health facility assessment data

Blood culture service readiness data were presented for each country using descriptive statistics (Stata 17, StataCorp LLC, Texas, USA). Missing data and adjusted denominators were indicated. Categorical variables were shown as the number and proportion of hospital neonatal units. Hospitals were grouped into three types based on country classifications, and linkage to the WHO level of newborn care [1] provided at the hospital: District [26], Secondary/Zonal/Regional, and Tertiary/National.

#### Tiered performance categorisation

We categorised hospital neonatal units into four performance tiers based on (i) laboratory availability (obtained from HFAs), (ii) microbiology availability (obtained from HFAs), and (iii) blood culture use for newborns prescribed antibiotics (data obtained from the NID). *Tier 1* hospitals were those with no laboratories. *Tier 2* hospitals had a laboratory but no microbiology service to perform blood cultures. *Tier 3* hospitals had a laboratory and blood culture service, but blood culture use for newborns prescribed antibiotics was < 50%. *Tier 4* hospitals were those with > 50% blood culture use for newborns on antibiotics.

## Results

**Fig. 2:**
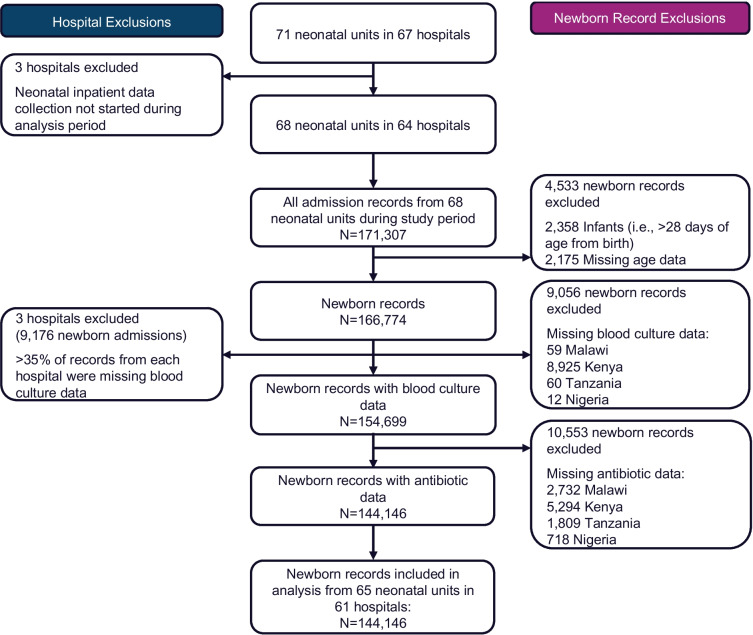
Flow diagram for the selection of hospitals and newborn records included in the study (January 2019–August 2022)

A flow diagram summarising the hospital and newborn record inclusion process is shown in Fig. 2. Between July 2019 and August 2022, 171,307 newborn records were reviewed, no duplicate records were found, and 4,533 (3%) records were excluded based on eligibility criteria. Of the 166,774 newborn records available for analysis, 9,056 (5%) and 10,553 (6%) were excluded due to missing blood culture and antibiotic data, respectively. Of those excluded, 14,219 (73%) were from Kenya (not all NID variables were part of routine data collection for hospitals in Kenya). Six hospitals were excluded based on eligibility criteria: three because neonatal inpatient data collection had not started at the hospitals during the analysis period, and three hospitals in Kenya which had > 35% records missing blood culture data. The remaining 144,146 newborn records from 65 neonatal units in 61 hospitals were included in the study.

Of the 65 units included, there were 10 in Kenya, 37 in Malawi, 11 in Nigeria, and seven in Tanzania (Table 1). Almost all hospitals (91%) were in urban/semi-urban areas. All hospitals in Nigeria and Tanzania, and the majority in Kenya (90%), were Secondary/Zonal/Regional or Tertiary/National hospitals. In Malawi, 65% were District hospitals [26]. Inpatient data collection at each hospital ranged from 16–22 months.

**Table 1:** Distribution of neonatal units implementing with NEST360 in Kenya, Malawi, Nigeria, and Tanzania

	**Kenya**		**Malawi**		**Nigeria**		**Tanzania**		**Total**	
**Neonatal Units**^a^** with NEST360 (*n* = 65)**	**10**		**37**		**11**		**7**		**65**	
	**n**	**(%)**	**n**	**(%)**	**n**	**(%)**	**n**	**(%)**	**n**	**(%)**
**Hospital Types**^b^										
District^c^	1	(10)	33	(90)	0	(0)	0	(0)	34	(52)
Secondary/Regional/Zonal	9	(90)	2	(5)	6	(55)	5	(71)	22	(33)
Tertiary/National	0	(0)	2	(5)	5	(46)	2	(29)	9	(134)
**Urban/Rural**^d^										
Urban	3	(30)	30	(81)	11	(100)	7	(100)	51	(79)
Semi-urban	5	(50)	3	(8)	0	(0)	0	(0)	8	(12)
Rural	2	(20)	4	(11)	0	(0)	0	(0)	6	(9)
**Median months with NEST360 (IQR)**	22 (5)		22 (8)		16 (7)		18 (67)		21.5 (8)	

^a^Some hospitals have more than one neonatal unit, especially in Nigeria (e.g., inborn and outborn units)

^b^Some variation in categorisation exists between countries

^c^Includes some faith-based hospitals (e.g., Christian Health Association of Malawi (CHAM) facilities)

^d^Defined by *Degree of Urbanisation* method, World Bank [27]

Note: Hospitals began prospective data collection at different time points. The median number of months of implementation is presented in the table

*Abbreviations*: *IQR* Inter-quartile range

### Objective 1: detection gap

**Fig. 3:**
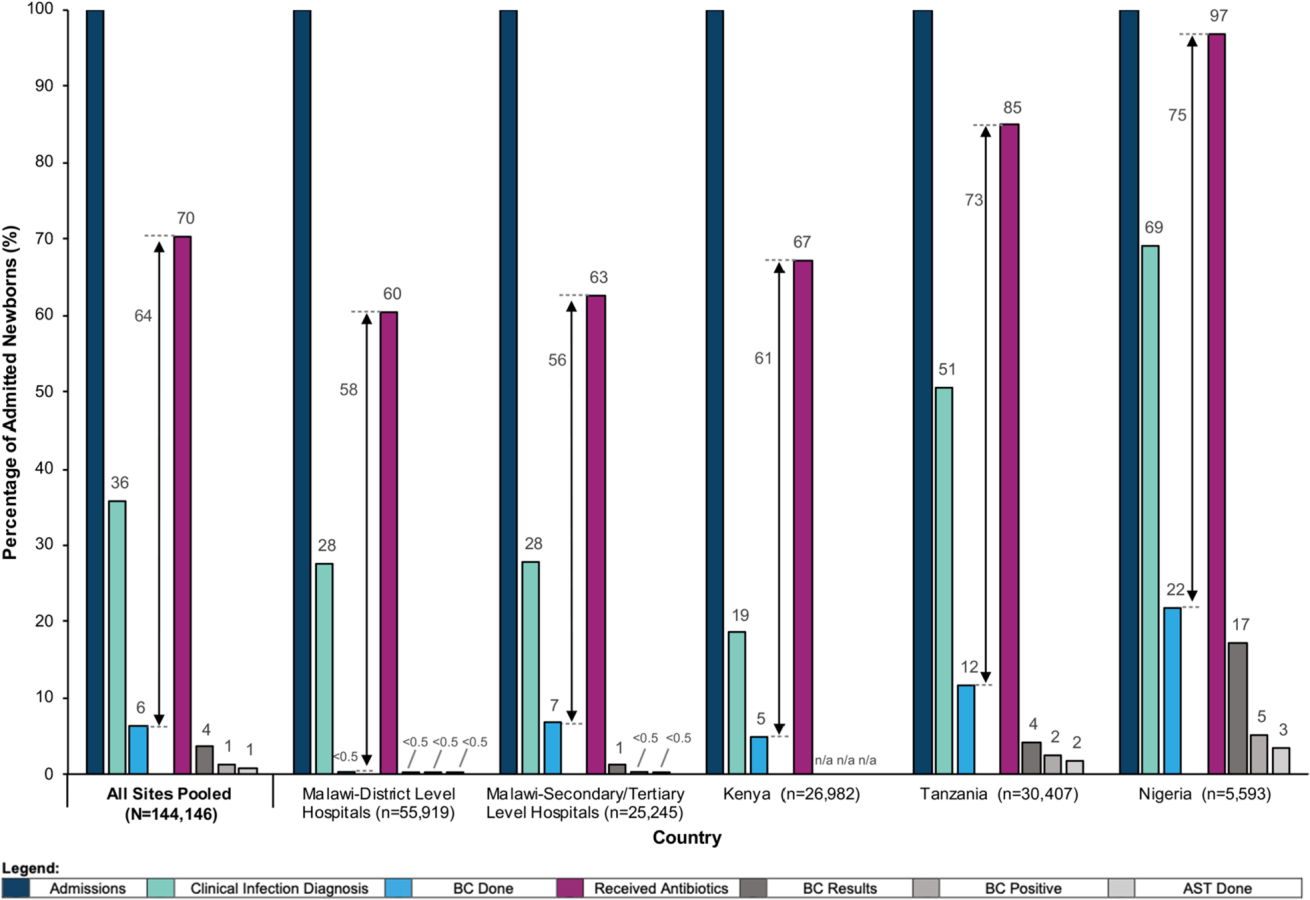
Neonatal infection detection and care gaps for countries implementing with NEST360; Kenya, Malawi, Nigeria, and Tanzania (*N* = 65 neonatal units and *N* = 144,146 newborn records), January 2019 – August 2022. Abbreviations: BC; blood culture, AST; antimicrobial sensitivity testing, n/a; not applicable. Note: Estimates are reported as pooled hospital means

Figure 3 presents neonatal infection detection and care gaps overall and by country. Overall, one-third (36%, 95%CI 30–41) of newborns had clinically diagnosed infections. Antibiotics were prescribed for 70% (65–75), but only 6% (3–10) had a blood culture. Documented blood culture results were even lower (4%, 1–6), with further drop-offs for positive blood cultures (1%, 0–2) and cultures with antimicrobial sensitivity testing results (1%, 0–1).

Despite a neonatal infection rate of 28% (24–31), District hospitals in Malawi performed very few blood cultures for newborns (0%, 0–1). Even though blood culture use was higher in Tertiary/National hospitals in Malawi, the gap between blood culture use and antibiotic prescription (57%) was similar to District hospitals in Malawi (58%). Neonatal blood culture use was second lowest in Kenya (5%, 0–11) but with the lowest clinically diagnosed infection rates for neonatal inpatients (19%, 12–25). Half of the admitted newborns in Tanzania were clinically diagnosed with an infection. However, the proportion of inpatients prescribed antibiotics and investigated with a blood culture was 85% (73–97) and 12% (0–30), respectively. Nigeria had the highest blood culture use (22%, 8–36), but almost all (97%, 93–100) newborns were prescribed antibiotics, resulting in the largest gap across all countries (73%).

**Fig. 4:**
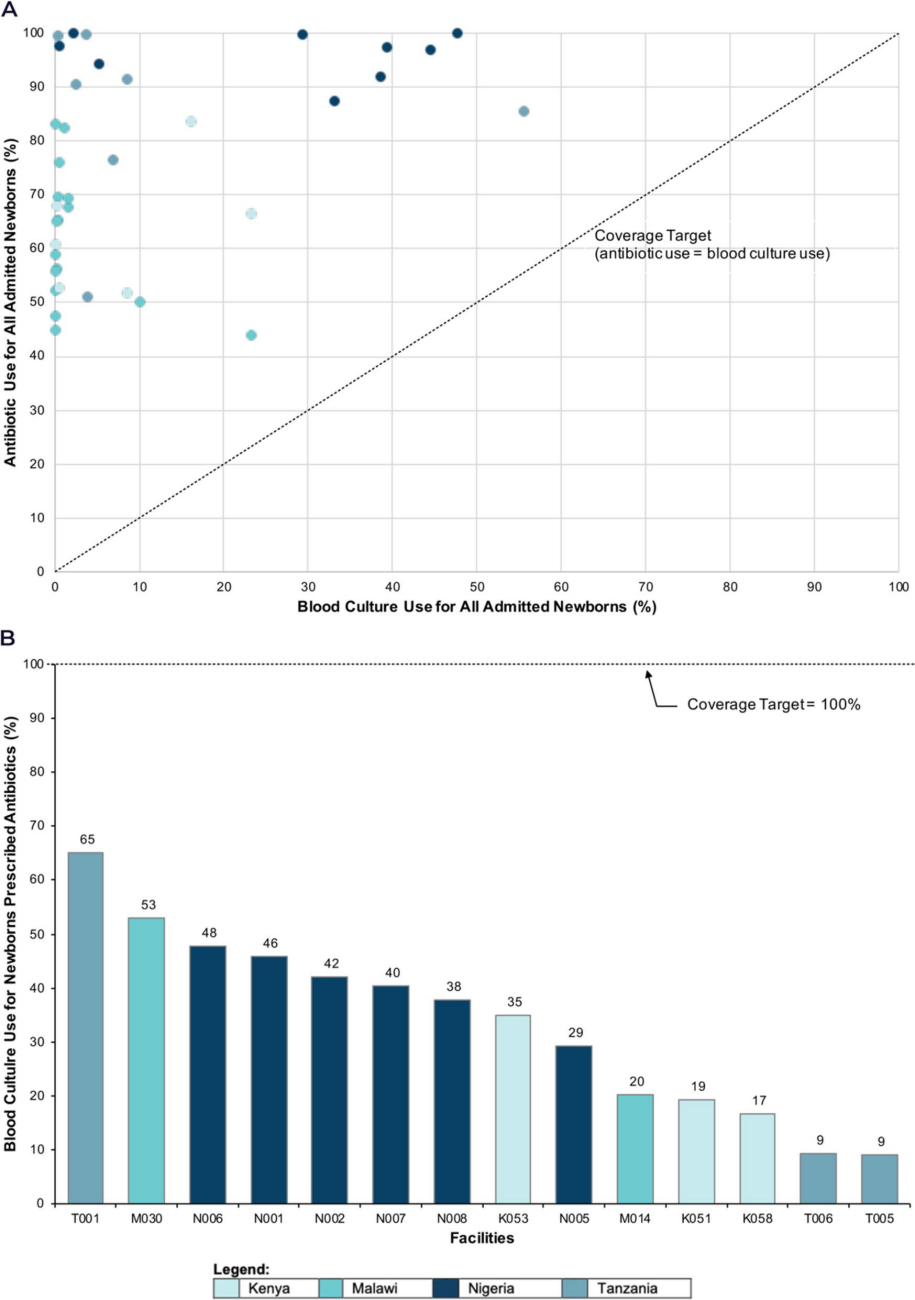
Neonatal unit blood culture use and antibiotic prescription with positive outliers for infection detection. **a** Scatter plot depicting blood culture use and antibiotic prescription per neonatal unit, as a percentage of total neonatal admissions. Each dot represents a neonatal unit. **b** Ranked positive outlier neonatal units* based on blood culture use for those newborns prescribed antibiotics during admission. *Positive outlier neonatal units are those with blood culture use, for newborns prescribed antibiotics, above the pooled hospital mean

Antibiotic prescribing and blood culture use varied across neonatal units (Fig. 4a). Blood culture use ranged from 0% to 57%. Antibiotic prescribing ranged from 44% to 100%. No unit met the best practice target of matched antibiotic and blood culture use. Fifteen neonatal units were positive outliers (i.e., blood culture use for those newborns prescribed antibiotics was higher than the pooled mean of 8%) (Fig. 4b). The highest-performing neonatal unit was in Tanzania, with blood culture use of 65% (1,179/1,813). The highest-performing neonatal units in Malawi, Nigeria, and Kenya had blood culture use of 53% (2,580/4,870), 48% (529/1,109), and 35% (1,208/3,456), respectively. Blood culture use was < 1% at 42/65 neonatal units (65%), and 40% (26/65) did no cultures. Blood culture use per neonatal unit is summarised in Additional file 5.

### Objective 2: hospital performance categorisation

**Fig. 5:**
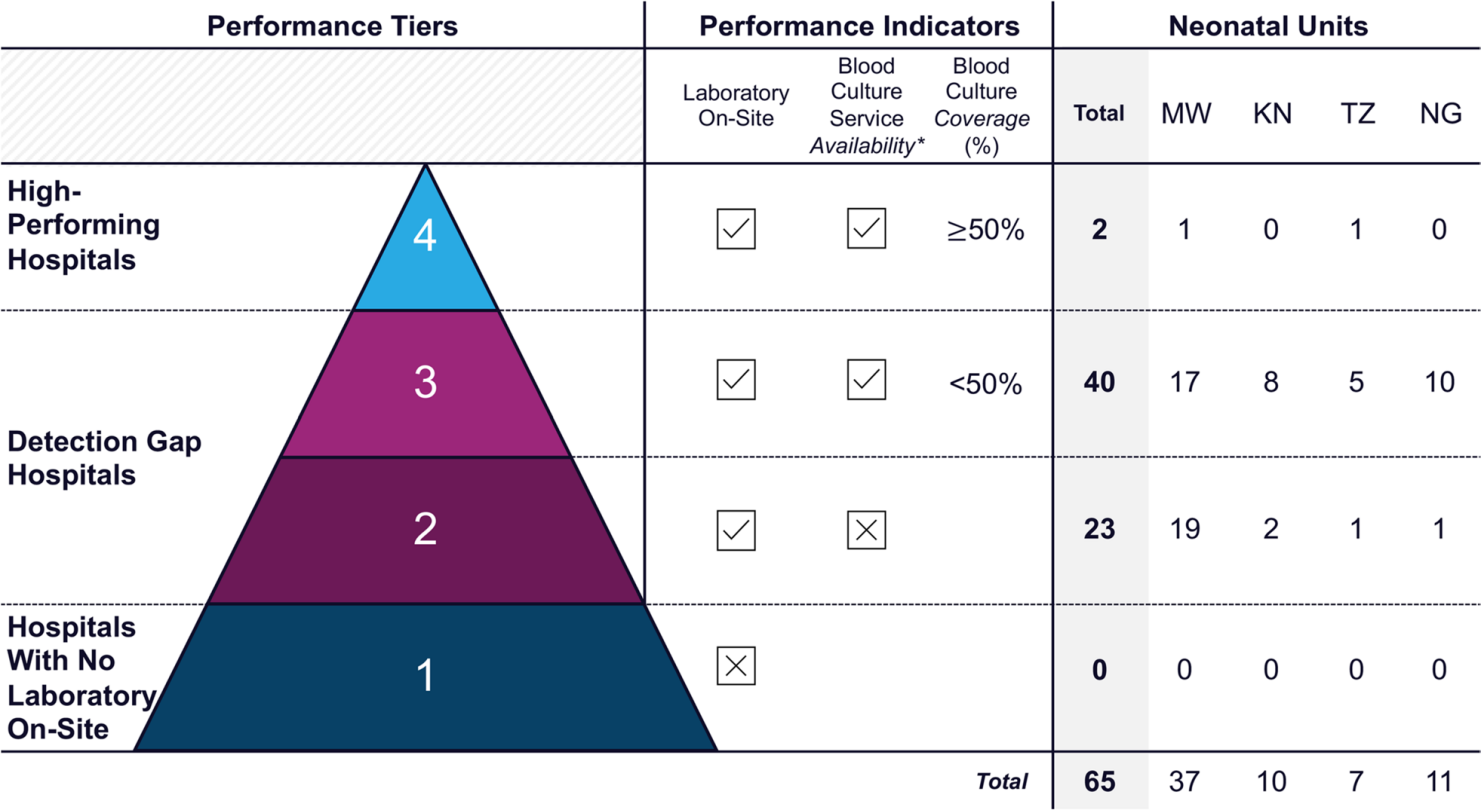
Tiered performance categorisation of hospitals implementing with NEST360 based on neonatal blood culture service availably and use (*N* = 65 neonatal units). *Defined as reported ability to perform blood culture at baseline Health Facility Assessment collection timepoint. Abbreviations: KN; Kenya, MW; Malawi, NG; Nigeria, TZ; Tanzania

The tiered categorisation of hospital neonatal units by blood culture performance is shown in Fig. 5. There were no *Tier 1* hospitals, as all had a laboratory. Twenty-two hospitals had a laboratory but could not do microbiological blood cultures (*Tier 2*). Most neonatal units were *Tier 3* (63%); units where < 50% of newborns on antibiotics had a blood culture, despite culture service availability. Only two hospitals with blood culture services had > 50% blood culture use for newborns prescribed antibiotics (*Tier 4*).

Laboratory and neonatal unit readiness to perform neonatal blood culture, and general hospital requirements for culture, are presented in Table 2.

**Table 2:** Hospital capacity to perform neonatal blood culture in ward and laboratory settings across Kenya, Malawi, Tanzania, and Nigeria (*n* = 65 neonatal units)

	**Kenya**		**Malawi**		**Nigeria**		**Tanzania**		**Total**	
**Neonatal Units with NEST360 (*n* = 65)**	**10**		**37**		**11**		**7**		**65**	
	**n**	**(%)**	**n**	**(%)**	**n**	**(%)**	**n**	**(%)**	**n**	**(%)**
**Laboratory Readiness for Culture**										
Total hospitals with a laboratory	10	(100)	37	(100)	11	(100)	7	(100)	65	(100)
Laboratory service available 24/7	10	(100)	36	(97)	10	(91)	7	(100)	63	(97)
Laboratory can do Gram staining	10	(100)	33	(89)	9	(82)	6	(86)	58	(89)
Laboratory can perform cultures on samples of blood^a^	9	(90)	18	(49)	10	(91)	7	(100)	44	(69)
* Using Automated Methods*	3	(33)	-		2	(20)	4	(57)	9	(21)
* Using Manual Methods*	2	(22)	-		5	(50)	1	(14)	8	(18)
* Not Asked*	4	(44)	18	(100)	3	(30)	3	(43)	28	(64)
Laboratory can perform AST on samples of blood^a^	7	(70)	3	(10)	8	(73)	5	(71)	23	(4)
**Commodities**										
Microscope	10	(100)	37	(100)	4	(36)	7	(100)	58	(89)
Blood culture bottle (paediatric)^b^	3	(38)	2	(7)	9	(82)	5	(71)	19	(35)
Blood culture bottle (adult)^b^	3	(38)	1	(3)	8	(73)	5	(71)	17	(31)
Petri dishes	9	(90)	18	(49)	9	(82)	7	(100)	43	(66)
Culture media^b^	7	(88)	10	(35)	9	(82)	7	(100)	30	(55)
Sterile picks and loops^b^	7	(88)	17	(59)	9	(82)	7	(100)	40	(73)
**Protocols**										
Protocol for reporting culture results back to neonatal unit^b,c^	1	(11)	3	(17)	7	(70)	5	(71)	16	(36)
**Neonatal Unit Readiness for Culture**										
**Human Resources**										
Nurse:newborn ratio (day of HFA visit)	1:6		1:11		1:11		1:2		1:7	
Nurse:newborn ratio (night before HFA visit)	1:12		1:19		1:15		1:4		1:12	
**Commodities**										
Antiseptics	10	(100)	33	(89)	7	(64)	7	(100)	57	(88)
Gloves^b^	8	(100)	29	(100)	6	(55)	5	(71)	48	(87)
Gauze	9	(90)	36	(97)	7	(64)	7	(100)	59	(91)
Sterile needles or butterfly set	9	(90)	20	(54)	6	(55)	2	(29)	59	(91)
Sterile syringe (any size)	10	(100)	34	(92)	7	(64)	7	(100)	58	(89)
**Protocols**										
Protocol for early diagnosis and management of neonatal infection^b^	8	(100)	18	(62)	7	(64)	7	(100)	40	(73)
Protocol for receiving results from lab & adding to patient files^b^	1	(13)	11	(38)	10	(91)	5	(71)	27	(49)
**General Facility Requirements**										
**Electrical Power Access**										
Connected to central electricity grid	10	(100)	37	(100)	8	(73)	6	(86)	61	(94)
Backup power cover for neonatal unit (i.e., generator, solar, battery inverter)	10	(100)	34	(92)	9	(82)	7	(100)	60	(92)
**Water Access**										
Borehole (electric)	2	(20)	7	(19)	5	(46)	2	(29)	16	(25)
Piped from municipality	8	(80)	28	(76)	5	(46)	4	(57)	45	(69)
Other	0	(0)	2	(5)	1	(9)	1	(14)	4	(6)

^a^Question not asked at eight hospitals in Malawi

^b^Question not asked at eight hospitals in Malawi and two hospitals in Kenya

^c^Only asked if hospital reported ability to perform cultures on blood samples

"-" = Not applicable

Note: Definitions of each row variable are provided in Additional file 3

*Abbreviations*: *24/7* 24 hours a day, seven days a week, *AST* Antimicrobial sensitivity testing, *HFA* Health Facility Assessment

#### Laboratory readiness

Most laboratories provided a 24-hour laboratory service (97%). All hospitals had a laboratory, with > 90% doing blood cultures in Kenya, Nigeria, and Tanzania. In Malawi (which had the greatest proportion of District hospitals), 49% of hospitals performed blood cultures. Most laboratories performed Gram staining (89%). Manual blood culture methods were used in half of the laboratories in Nigeria compared to automated systems in 57% of laboratories in Tanzania. More than 70% of laboratories in Kenya, Nigeria and Tanzania did antimicrobial sensitivity testing; only 10% in Malawi.

Half of the laboratories had microscopes, Petri dishes, culture media, and sterile picks and loops. Only a third had blood culture bottles (i.e., paediatric or adult). Blood culture bottle availability in laboratories varied between countries (3–82%), with only marginal differences in paediatric (35%) and adult (31%) blood culture bottle availability across sites. Most laboratories in Nigeria and Tanzania had protocols for reporting culture results to neonatal units (70% and 71%, respectively). Only 11% and 17% of laboratories in Kenya and Malawi had these protocols, respectively.

#### Neonatal unit readiness

There was wide inter-country variation in nurse-to-newborn ratios on the day of the HFA (1:2 in Tanzania vs 1:11 in Malawi and Nigeria). Staffing ratios were lower on the night shift before the HFA, ranging from 1:4 to 1:19. Neonatal units were generally well stocked with consumables for sterile blood sample collection. A protocol for ‘early diagnosis and management of neonatal infection’ was available in all Kenya and Tanzania units. Only 60% of Malawi and Nigeria units had such protocols. Units with a protocol for ‘receiving and documenting laboratory results in patients’ files’ ranged from 13% in Malawi to 91% in Nigeria.

#### General hospital requirements for culture

Most hospitals were connected to a central electricity grid (94%), and 93% had a backup power source for the neonatal units (i.e., generator, solar power, or battery inverter). All hospitals had water access. Municipal piped water was the primary source for Kenyan, Malawian, and Tanzanian hospitals. An equal number of hospitals in Nigeria used piped water and electric borehole supply.

## Discussion

The standard of care for clinically-defined neonatal infections is diagnostic blood culture and antibiotic treatment [14, 16]. This large, multi-country analysis of the gap between antibiotic prescribing and blood culture use for newborns, using routine data, revealed that for 144,146 newborns admitted across 61 hospitals, most were prescribed antibiotics (70%, 95%CI 65–75), but very few had blood cultures (6%, 95%CI 3–10). Neonatal unit blood culture use for newborns prescribed antibiotics varied (0–65%), and none met the quality-of-care target of ~ 100% [16]. Positive outlier hospitals in each country demonstrate that performing more neonatal blood cultures is possible in LMIC hospital settings. However, there is a major missed opportunity for neonatal infection detection in two-thirds of hospitals where laboratories with blood culture services are available, but culture use is < 50%. Hospitals with laboratories have an opportunity to implement quality improvement strategies to increase blood culture usage and improve newborn outcomes.

### Blood culture gap and hospital performance

Neonatal blood culture use was markedly low overall. Non-utilisation of culture in the diagnosis of neonatal sepsis can have significant implications on diagnostic accuracy, leading to delayed or missed treatment and potentially negative outcomes. Blood culture remains the diagnostic standard for neonatal sepsis, enabling identification of causative agents and selection of targeted antibiotics. Empiric treatment without blood culture can lead to unnecessary antibiotic exposure and increased AMR. Despite low culture use overall, there was some variation across hospitals, consistent with other multi-site studies [19]. A lack of basic laboratory infrastructure did not account for low routine culture use in this study, as no hospitals were without laboratories (*Tier 1*). Neonatal infection prevention and care bundles for LMICs contain very few detection elements, possibly exacerbating the detection gap in NEST360-associated neonatal units [28].

Most hospitals with laboratories but no microbiology (*Tier 2*) were in Malawi (19/22, 86%), despite the country’s efforts in recent years to improve institutional infrastructure and AMR policy [29]. Almost all these hospitals were District hospitals (17/19, 89%), the level at which blood culture should ideally be available [30]. The positive outlier hospital in Malawi has support from international research institutions to undertake onsite microbiological surveillance [29]. This Tertiary/National hospital accounted for 91% (2,580/2,847) of all cultures across the 37 Malawian hospitals, indicating that national AMR surveillance is limited to a single sentinel site. It also highlights the gap between Malawi's Tertiary/National and District hospital laboratory capacity. Health system factors that might exacerbate this gap include understaffing, and unmet training and accreditation needs in District laboratories, especially as countries expand neonatal care at the district level to meet ENAP target 4 and SDG 3.2 [31].

Laboratories with microbiology were available for most neonatal units across the countries included in this study (43/65, 66%). However, almost all performed < 50% cultures for newborns on antibiotics (41/43, 95%), supporting evidence that laboratory services remain underutilised even when diagnostic testing is available (*Tier 3*) [32]. Consumable and human resource constraints likely impact the blood culture service in these hospitals. For example, blood culture bottles and culture media were only available in 30% and 55% of hospitals, respectively, which would limit blood culture use. Additionally, in Africa, the availability of quality-assured consumables is often compromised by lack of onsite production and ineffective supply chains, which are incompatible with product shelf life and cold storage requirements [33–35]. Lack of culture bottles might also reflect insufficient resources available to purchase blood culture bottles, as well as various other microbiology laboratory supplies. There is also limited commercial interest in developing new diagnostics adapted for low-resource settings because of low profit margins [35]. Although, some manufacturers have launched research initiatives for low-cost innovations targeting simplified blood culture systems [36]. Insufficient neonatal unit staff might impede culture use, particularly at night when nurse-to-newborn ratios in this study decreased. Shortages and inequitable distribution of competent, motivated health workers result in heavy workloads for existing staff, unacceptable staffing ratios, and gaps in effective coverage of newborn care, including infection detection and management [37]. Human resource strategies to improve newborn care in health facilities in LMIC require prioritisation [38].

Who pays for laboratory tests likely contributes to the infection detection gap. Most health expenditures in LMIC rely on out-of-pocket payments by families. Studies in Nigeria show that costs for paediatric laboratory services are borne by families and are often barriers to access. [39]. Healthcare users tend to underinvest in diagnostic tests, perceiving more immediate value in treatment versus diagnosis [40]. These factors could contribute to fewer requests for blood cultures.

The Unitaid Fever Diagnostic Landscape points out that short reagent shelf life, supply chain difficulties, and need for highly skilled labour drive up the cost of blood cultures in LMIC, resulting in few blood cultures performed [41]. The low numbers further contribute to higher prices per test, creating a vicious cycle [42]. Governments aiming for universal healthcare coverage are unlikely to be able to cover these costs with their current designated health expenditure. For example, in Kenya, the cost of processing a single blood culture specimen (US$5.07 in 2017) constitutes a substantial proportion of per capita public expenditure on health from domestic sources (US$27.96 in 2017) [29, 43, 44].

Clinicians may deprioritise diagnostic testing for newborns with suspected infection, particularly if unreliability and prolonged turnaround times limit the utility of blood cultures in clinical decision-making. Studies have shown that clinicians in Kenyan referral hospitals often perceive diagnostic tests as unreliable or unhelpful, leading to the underutilisation of laboratory services [45, 46]. Blood culture utility may be hampered by limited communication between the laboratory and neonatal unit [35, 47, 48]. To address this issue, there is a need to increase demand for culture testing among clinicians and to evaluate the laboratory-ward interface, which is poorly studied in low-resource settings [30]. The interface could be strengthened by educating clinicians on laboratory principles [49–51] and improving laboratory management information systems, which facilitate communication of test results and improve AMR surveillance [52]. Our study showed that availability of protocols for ‘reporting results back to the neonatal unit’ and ‘adding laboratory results to patient records’ was low (36% and 49%, respectively). Effective and timely communication of test results is critical to ensure diagnostic culture is utilised to inform patient care.

### Antibiotic prescribing

Antibiotic prescribing was high in each hospital, with some neonatal units prescribing antibiotics to all admissions. Elevated antibiotic use is well-documented in neonatal inpatient settings, particularly in LMIC [19, 53, 54]. Such findings are unsurprising, as hospitalised newborns are especially vulnerable to infection and can rapidly deteriorate if infected, so clinical infection algorithms prioritise sensitivity over specificity [55]. The result is a tendency for clinicians to prescribe antibiotics as a safeguard against severe infection, implicitly calculating that the benefits outweigh the risks for the baby. In high-resourced settings, these patients would have a blood culture performed to inform antibiotic de-escalation or cessation. With limited availability or use of cultures, such decisions rely on clinical acumen alone, which can result in prolonged antibiotic therapy and hospitalisation, driving higher healthcare costs [56]. Excess antibiotic exposure can also have consequences for the newborn (including increased risk of necrotising enterocolitis [57], medication-related adverse events, infant-mother separation and an altered microbiome [58, 59]) and society (through the propagation of AMR) [60]. Improved antibiotic stewardship is imperative in every country and for every neonate but is improbable without increased diagnostic use in hospitals [61].

### Strengths and limitations

Our study has strengths, notably the large number of neonatal units and newborn records, standardised data collection tools across sites, and trained data collectors. Generalisability in Malawi is good since implementation with NEST360 is occuring nationally (i.e., in all public district, regional, and mission hospitals). Fewer hospitals are assiocated with NEST360 in Kenya, Tanzania, and especially a smaller proportion in Nigeria. Nonetheless, the results still provide insight into the extent of detection failure for newborn infections in the sub-Saharan African region and advocate strongly for improvement. The NID used for this study was systematically designed as a parsimonious tool focusing on critical indicators for impact. The tool supports frequent data quality assessments, ensuring consistent data use across sites to drive action. The opportunity for linkage with HFA data provided hospital-level service readiness context to individual-level data.

There were several limitations. First, these data are primarily from 2020–2021, and some factors may have changed after NEST360 implementation, such as infrastructure or staffing [62]. Second, certain data were not available, including the timing of blood sampling versus antimicrobial administration, antibiotic dose and duration (limiting quality and appropriateness assessment of antibiotic prescriptions), antibiogram availability, and laboratory access to backup power. NEST360 plans to analyse the association between antibiotic choice, bacterial pathogen identified by blood culture, and outcomes such as neonatal mortality and extended hospitalisation in future studies. Finally, results were only documented for half of the blood cultures performed across all hospitals. This lack of documentation may reflect poor case note documentation practices, a widely recognised issue even in well-resourced settings [63], or quality gaps in laboratory information management systems. Shifting toward electronic systems could improve result documentation, but managing extensive electronic databases is challenging [64, 65], and more complex systems are required to collect individually-linked microbiology data. Implementation research is underway to assess if case-based antibiotic resistance surveillance is feasible in LMIC [66].

The neonatal infection detection gap is actionable. Strategies to embed blood culture as routine standard of care for neonatal infection management require prioritisation by country governments, policymakers, clinicians, and laboratory staff. Short-term, efforts to improve culture use could focus on hospitals where laboratory and microbiology services are available but underutilised (*Tier 3*). This could be achieved through iterative performance feedback, training, and strong leadership [47, 53]. Longer-term, high-quality microbiology data needs to be collected, analysed, and disseminated to improve culture uptake, antimicrobial stewardship policies, and newborn outcomes. Availability of antibiograms at the hospital, regional, and country levels are required to appropriately target therapy. Using clinical audit data to stimulate quality improvement across hospitals with unexplained variation is a topical issue in high-income countries [67]. Hospitals implementing with NEST360 could be provided with NID-linked quality dashboards to aid quality improvement efforts [68]. Development of low-cost, point-of-care sepsis diagnostics could be transformative, but blood culture remains the current best practice [69, 70]. Additionally, even when available, there is substantial variability in use of POCTs for management of childhood infections across countries [71]. Newborns with infections, but who do not access hospital care, must not be forgotten (Fig. 1). Intersectoral efforts are necessary to address access and cost barriers to quality healthcare.

Future research could focus on why hospitals with similar laboratory and ward capacities vary in their routine use of laboratory diagnostics for newborns. A qualitative exploration of local barriers and enablers to blood culture is underway in hospitals implementing with NEST360. The clinical pathway encompassing the decision to perform blood culture, sample collection, specimen processing, and result communication involves various activities and stakeholders. Implementation research is required to identify and overcome local rate-limiting steps in these processes, especially for LMIC.

## Conclusion

Whilst major gaps between antibiotic and blood culture use for admitted newborns are present, there is potential to improve. In NEST360-associated hospitals, variation in blood culture use indicates that strategies for system strengthening can be locally targeted. Efforts should focus on increasing routine blood culture use and improving the laboratory-ward interface to reduce the burden of neonatal HCAIs and AMR, especially in hospitals with functional microbiological services. Strengthening laboratory capacity in district-level hospitals remains a priority as the world moves to scale care for small and sick newborns to meet SDG 3.2 and ENAP target 4.

## Supplementary Information

**Additional file 1.** National newborn statistics and data sources for the four countries implementing with NEST360. Selected newborn statistics demonstrating differences in SSNC requirements between countries (using national data from external sources).

**Additional file 2.** STROBE Checklist. Checklist of items that should be included in reports of observational studies.

**Additional file 3.** Variables included in the analysis. Listed in the table are variables by outcome, newborn characteristics, and hospital characteristics. The variable type is also provided, and an explanation as to how variables were transformed and why.

**Additional file 4.** Characteristics of eligible newborns admitted to hospitals implementing with NEST360 during the study period, January 2019–August 2022 (N=144,146 newborn records). Table of characteristics of newborn records included in the study, stratified by country.

**Additional file 5.** Blood culture use and antibiotic prescriptions per neonatal unit included in the study. Table with each row representing a neonatal unit included in the study, reporting total admissions, infections diagnoses, antibiotic prescriptions, and blood cultures done.

**Additional file 6.** Local ethical approval for the complex evaluation of the implementation of a small and sick newborn care package with NEST360. Table of country protocol titles and Local Ethics Committee protocol identity numbers.

## Data Availability

All collaborating partners in the NEST360 Alliance jointly developed and signed data sharing and transfer agreements. The NID and HFA tools, data dictionaries and training are available from NEST360/UNICEF Implementation Toolkit for Small and Sick Newborn Care [72].

## Abbreviations

AMR: Antimicrobial resistance
CI: Confidence interval
ENAP: Every Newborn Action Plan
HCAI: Healthcare-associated infection
HFA: Health Facility Assessment
LMIC: Low- and middle-income countries
NID: Neonatal Inpatient Dataset
NEST360: Newborn Essential Solutions and Technologies
SDGs: Sustainable Development Goals
SSNC: Small and sick newborn care
WHO: World Health Organization
