# Supplementary material for: Blood culture versus antibiotic use for neonatal inpatients in 61 hospitals implementing with the NEST360 Alliance in Kenya, Malawi, Nigeria, and Tanzania: a cross-sectional study
Source: BMC Pediatr. 2023 Nov 15;23(Suppl 2):568. doi: 10.1186/s12887-023-04343-0 (PMC10652421; doi:10.1186/s12887-023-04343-0)
Supplement: Supplementary file 1 — Additional file 1. National newborn statistics and data sources for the four countries implementing with NEST360. Selected newborn statistics demonstrating differences in SSNC requirements between countries (using national data from external sources). [file 12887_2023_4343_MOESM1_ESM.docx]

**Additional File 1. National newborn statistics and data sources for the four countries implementing with NEST360**

| **INDICATOR** | **Malawi** | **Kenya** | **Tanzania** | **Nigeria** |
| --- | --- | --- | --- | --- |
| Neonatal mortality rate per 1000 live births^a^ | 19.1 | 20.1 | 20.5 | 35.5 |
| Number of annual neonatal deaths^b^ | 12,225 | 30,853 | 43,285 | 270,705 |
| Preterm birth rate per 100 live births^c^ | 18 | 12 | 11 | 12 |
| Neonatal deaths due to sepsis^d^ | 18.6 | 15.8 | 19.7 | 16 |
| Births in a health facility (% of live births) | 91.4^e^ | 61.2^f^ | 62.6^g^ | 39.4^h^ |

**Data Sources:**

1. United Nations Interagency Group for Child Mortality Estimation (UN IGME). *Levels and trends in child mortality – 2021 Report.* Accessed 4 November 2022. Available: <<https://data.unicef.org/resources/levels-and-trends-in-child-mortality/>>.
2. World Bank. *Number of neonatal deaths 2020*. Accessed 4 November 2022. Available: <<https://data.worldbank.org/indicator/SH.DTH.NMRT?locations=KE>>.
3. Blencowe H, Cousens S, Oestergaard M, Chou D, Moller AB, Narwal R, Adler A, Garcia CV, Rohde S, Say L, Lawn JE. National, regional and worldwide estimates of preterm birth rates in the year 2010 with time trends since 1990 for selected countries: a systematic analysis and implications. *The Lancet*, June 9 2012, 379(9832): 2162-72.
4. United Nations Inter-agency Group for Child Mortality Estimation (UN IGME). *Levels and trends in child mortality – 2016 Report.* Accessed 4 November 2022. Available: <<https://data.unicef.org/resources/levels-and-trends-in-child-mortality/>>.
5. Malawi Demographic and Health Survey 2015-2016 via the DHS Program STATcompiler. (http://www.statcompiler.com).
6. Kenya Demographic and Health Survey 2014 via the DHS Program STATcompiler. (<http://www.statcompiler.com>).
7. United Republic of Tanzania Demographic and Health Survey 2015-2016 via the DHS Program STATcompiler. (<http://www.statcompiler.com>).
8. Nigeria Demographic and Health Survey 2018 via the DHS Program STATcompiler. (http://www.statcompiler.com).

## Additional File 2. *STROBE checklist*

*Checklist of items that should be included in reports of observational studies.*

|  | Item No. | Recommendation | Page  No. |
| --- | --- | --- | --- |
| **Title and abstract** | 1 | (*a*) Indicate the study’s design with a commonly used term in the title or the abstract |  |
|  |  | (*b*) Provide in the abstract an informative and balanced summary of what was done and what was found |  |
| Introduction | | | |
| Background/rationale | 2 | Explain the scientific background and rationale for the investigation being reported |  |
| Objectives | 3 | State specific objectives, including any prespecified hypotheses |  |
| Methods | | | |
| Study design | 4 | Present key elements of study design early in the paper |  |
| Setting | 5 | Describe the setting, locations, and relevant dates, including periods of recruitment, exposure, follow-up, and data collection |  |
| Participants | 6 | (*a*) *Cohort study*—Give the eligibility criteria, and the sources and methods of selection of participants. Describe methods of follow-up  *Case-control study*—Give the eligibility criteria, and the sources and methods of case ascertainment and control selection. Give the rationale for the choice of cases and controls  *Cross-sectional study*—Give the eligibility criteria, and the sources and methods of selection of participants |  |
|  |  | (*b*) *Cohort study*—For matched studies, give matching criteria and number of exposed and unexposed  *Case-control study*—For matched studies, give matching criteria and the number of controls per case |  |
| Variables | 7 | Clearly define all outcomes, exposures, predictors, potential confounders, and effect modifiers. Give diagnostic criteria, if applicable |  |
| Data sources/ measurement | 8* | For each variable of interest, give sources of data and details of methods of assessment (measurement). Describe comparability of assessment methods if there is more than one group |  |
| Bias | 9 | Describe any efforts to address potential sources of bias |  |
| Study size | 10 | Explain how the study size was arrived at |  |
| Quantitative variables | 11 | Explain how quantitative variables were handled in the analyses. If applicable, describe which groupings were chosen and why |  |
| Statistical methods | 12 | (*a*) Describe all statistical methods, including those used to control for confounding |  |
|  |  | (*b*) Describe any methods used to examine subgroups and interactions |  |
|  |  | (*c*) Explain how missing data were addressed |  |
|  |  | (*d*) *Cohort study*—If applicable, explain how loss to follow-up was addressed  *Case-control study*—If applicable, explain how matching of cases and controls was addressed  *Cross-sectional study*—If applicable, describe analytical methods taking account of sampling strategy |  |
|  |  | (*e*) Describe any sensitivity analyses |  |

*Continued on next page*

| **Results** |  |  |  |
| --- | --- | --- | --- |
| Participants | 13* | (a) Report numbers of individuals at each stage of study—eg numbers potentially eligible, examined for eligibility, confirmed eligible, included in the study, completing follow-up, and analysed |  |
|  |  | (b) Give reasons for non-participation at each stage |  |
|  |  | (c) Consider use of a flow diagram |  |
| Descriptive data | 14* | (a) Give characteristics of study participants (eg demographic, clinical, social) and information on exposures and potential confounders |  |
|  |  | (b) Indicate number of participants with missing data for each variable of interest |  |
|  |  | (c) *Cohort study*—Summarise follow-up time (eg, average and total amount) |  |
| Outcome data | 15* | *Cohort study*—Report numbers of outcome events or summary measures over time |  |
|  |  | *Case-control study—*Report numbers in each exposure category, or summary measures of exposure |  |
|  |  | *Cross-sectional study—*Report numbers of outcome events or summary measures |  |
| Main results | 16 | (*a*) Give unadjusted estimates and, if applicable, confounder-adjusted estimates and their precision (eg, 95% confidence interval). Make clear which confounders were adjusted for and why they were included |  |
|  |  | (*b*) Report category boundaries when continuous variables were categorised |  |
|  |  | (*c*) If relevant, consider translating estimates of relative risk into absolute risk for a meaningful time period |  |
| Other analyses | 17 | Report other analyses done—eg analyses of subgroups and interactions, and sensitivity analyses |  |
| **Discussion** |  |  |  |
| Key results | 18 | Summarise key results with reference to study objectives |  |
| Limitations | 19 | Discuss limitations of the study, taking into account sources of potential bias or imprecision. Discuss both direction and magnitude of any potential bias |  |
| Interpretation | 20 | Give a cautious overall interpretation of results considering objectives, limitations, multiplicity of analyses, results from similar studies, and other relevant evidence |  |
| Generalisability | 21 | Discuss the generalisability (external validity) of the study results |  |
| **Other information** |  |  |  |
| Funding | 22 | Give the source of funding and the role of the funders for the present study and, if applicable, for the original study on which the present article is based |  |

## Additional File 3: *Variables included in the analysis.*

Listed in the table are variables by outcome, newborn characteristics, and hospital characteristics. The variable type is also provided, and an explanation as to how variables were transformed and why.

| **OUTCOME VARIABLES (NID)** | | |
| --- | --- | --- |
| VARIABLE | TYPE | VARIABLE TRANSFORMATION |
| 1. Antibiotic prescribed | Binary | Collected as a binary variable in NID: “yes”; “no”. If “yes”, the antibiotic type was selected from a given list or provided as free text. |
| 2. Blood culture documented | Binary | Collected as a categorical variable in NID: “blood culture done – result unknown”; “blood culture done – result positive”; “blood culture done – result negative”; “blood culture not done”. Recoded into a binary variable as “blood culture done” and “blood culture not done.” |
| **NEWBORN BACKGROUND CHARACTERISTICS (NID)** | | |
| VARIABLE | TYPE | VARIABLE TRANSFORMATION |
| 1. Age at admission (days) | Categorical | Calculated by subtracting date of admission from date of birth and converting the value into a continuous variable with days as units. This categorical variable was then categorised into five age categories: <1, 1-3, 4-6, 7-13 and 14+. All negative intervals were recoded to missing. Most newborn admissions last between 0-3 days which is why this interval was split. |
| 2. Gestational age at admission (weeks) | Categorical | Collected as a continuous variable and categorised into four groups^62^: extremely preterm (less than 28 weeks); very preterm (28 to 32 weeks); moderate to late preterm (32 to 37 weeks), and term (37 weeks or more). |
| 3. Birth weight (g) | Categorical | Collected as a continuous variable and categorised bands to reflect internationally recognised birth weight definitions: very low birth weight <1500g; low birth weight 1500-2499g, normal birth weight 2500-3500g, high birth weight >4000g. |
| 4. Length of admission  (days) | Categorical | Calculated by subtracting date of admission from date of discharge and converting the value into a continuous variable with days as units. This categorical variable was then categorised into six intervals; 1-2 days, 3-13 days, and 14+ days to achieve a reasonable distribution of newborns across groups. All negative intervals were recoded as missing. |
| 5. Sex | Binary | Collected and coded as male, female, or indeterminate. |
| 6. Place of birth | Categorical | Collected as “inborn” or “outborn”. If “outborn”, either” at home”, “in transit”, “at a religious building”, or “another healthcare facility” was specified. Responses were recoded and collapsed into three categories: “born at facility”, “born at another facility”, “born at home”, the latter including “in transit”, and “at a religious building”. |
| 7. Condition at discharge | Binary | Collected and coded as alive or dead. |
| **HOSPITAL CHARACTERISTICS (HFA)** | | |
| VARIABLE | DESCRIPTION | |
| **Laboratory Readiness for Culture** | | |
| Laboratory service available 24/7 | Laboratory is operational 24 hours per day 7 days per week. This does not necessarily mean that the lab is staffed at all times. | |
| Laboratory can do Gram staining | Response options include Available today / Usually available, but not today / Not available, but can be outsourced / Not available today | |
| Laboratory can perform cultures on samples of blood |  |  |
| Laboratory can perform AST on samples of blood |  |  |
| Microscope | Response options include Available today and no stockout in last 4 weeks / Available today, but stockout in last 4 weeks / Not available today | |
| Blood culture bottle (paediatric) |  |  |
| Blood culture bottle (adult) |  |  |
| Petri dishes |  |  |
| Culture media |  |  |
| Sterile picks and loops |  |  |
| Microscope |  |  |
| Protocol for reporting culture results back to neonatal unit | All protocols must be observed by data collector in order to select yes. Response options include Available and easily accessible / Available, but not easily accessible / Not available. (This response is from the perspective of the lab manager/technician). | |
| **Neonatal Unit Readiness for Culture** | | |
| Nurse:newborn ratio (day of HFA visit) | Number of nurses providing exclusive care on the neonatal unit on the day of HFA visit to number of babies in the neonatal unit at the time of the HFA visit | |
| Nurse:newborn ratio (night before HFA visit) | Number of nurses providing exclusive care on the neonatal unit the night before HFA visit to number of babies in the neonatal unit at the time of the HFA visit | |
| Antiseptics | Response options include Available today and no stockout in last 4 weeks / Available today, but stockout in last 4 weeks / Not available today | |
| Gloves |  |  |
| Gauze |  |  |
| Sterile needles or butterfly set |  |  |
| Sterile syringe (any size) |  |  |
| Protocol for early diagnosis and management of neonatal infection | All protocols must be observed by data collector in order to select yes. Response options include Available and easily accessible / Available, but not easily accessible / Not available. (Response from the perspective of the neonatal unit nurse in charge). | |
| Protocol for receiving results from lab & adding to patient files |  |  |
| **General Facility Requirements** | | |
| Connected to central electricity grid | If yes, facility is connected to central electricity grid even if connection to electricity grid is irregular. | |
| Generator cover on neonatal ward | Facility backup power covers the neonatal unit even if not all lighting or equipment are covered by backup power. | |
| Water access | Options include piped water from municipality, borehole (either hand pump or solar pump), other (well (hand pump, electric, or solar), river) | |

**Abbreviations:** NID, neonatal inpatient dataset; HFA, health facility assessment, AST; antimicrobial sensitivity testing.

## Additional File 4: *Characteristics of eligible newborns admitted to hospitals implementing NEST360 during the study period, January 2019-August 2022* (N=144146 newborn records)

|  | **Malawi** |  | **Kenya** |  | **Tanzania** |  | **Nigeria** |  |
| --- | --- | --- | --- | --- | --- | --- | --- | --- |
| **Total Admissions** | **50256** |  | **19688** |  | **19213** |  | **2713** |  |
|  | n | (column %) | n | (column %) | n | (column %) | n | (column %) |
| **Age at admission** |  |  |  |  |  |  |  |  |
| <1 day | 36691 | (73.0) | 15870 | (80.6) | 12326 | (64.2) | 1326 | (48.9) |
| ≥1 to <4 days | 8308 | (16.5) | 3092 | (15.7) | 4505 | (23.4) | 784 | (28.9) |
| ≥4 to <7 days | 1211 | (2.4) | 381 | (1.9) | 757 | (3.9) | 246 | (9.1) |
| ≥7 to <14 days | 1922 | (3.8) | 254 | (1.3) | 848 | (4.4) | 175 | (6.5) |
| ≥14 days | 2124 | (4.2) | 91 | (0.5) | 777 | (4.0) | 182 | (6.7) |
| **Gestational age at admission** |  |  |  |  |  |  |  |  |
| Term (≥37 weeks) | 19333 | (44.4) | 9651 | (54.4) | 9680 | (61.1) | 641 | (35.8) |
| Late preterm (≥32 to <37 weeks) | 18081 | (41.5) | 4546 | (25.6) | 4560 | (28.8) | 646 | (36.0) |
| Very preterm (≥28 to <32 weeks) | 4226 | (9.7) | 2203 | (12.4) | 2205 | (13.9) | 362 | (20.2) |
| Extremely preterm (<28 weeks) | 1889 | (4.3) | 1351 | (7.6) | 1353 | (8.5) | 143 | (8.0) |
| Missing | 6727 |  | 1937 |  | 3365 |  | 921 |  |
| Median (IQR) |  |  |  |  |  |  |  |  |
| **Birth weight** |  |  |  |  |  |  |  |  |
| <1,000g (i.e., ELBW) | 690 | (1.4) | 598 | (3.0) | 438 | (2.3) | 100 | (3.7) |
| 1,000-1499g (i.e., VLBW) | 3399 | (6.8) | 1737 | (8.8) | 1416 | (7.4) | 223 | (8.2) |
| 1,500-2,499g (i.e., LBW) | 14234 | (28.3) | 5372 | (27.3) | 5680 | (29.6) | 619 | (22.8) |
| 2,500-3,499g | 22903 | (45.6) | 8035 | (40.8) | 8228 | (42.8) | 759 | (28.0) |
| 3,500+g | 9028 | (18.0) | 3946 | (20.0) | 3451 | (18.0) | 1012 | (37.3) |
| Missing | 2 |  | 0 |  | 0 |  | 0 |  |
| Mean (95%CI) |  |  |  |  |  |  |  |  |
| **Length of admission** |  |  |  |  |  |  |  |  |
| <1 day | 3636 | (7.8) | 796 | (4.0) | 789 | (4.1) | 139 | (5.1) |
| ≥1 to <3 days | 18024 | (38.9) | 5293 | (26.9) | 6162 | (32.1) | 364 | (13.4) |
| ≥3 to <7 days | 16872 | (36.4) | 6744 | (34.3) | 7398 | (38.6) | 873 | (32.2) |
| ≥7 to <14 days | 5116 | (11.0) | 3479 | (17.7) | 3016 | (15.7) | 883 | (32.6) |
| ≥14 days | 2720 | (5.9) | 3366 | (17.1) | 1802 | (9.4) | 453 | (16.7) |
| Missing | 3888 |  | 10 |  | 46 |  | 1 |  |
| Mean (95%CI) |  |  |  |  |  |  |  |  |
| Female | 20331 | (44.8) | 8418 | (42.9) | 8608 | (44.9) | 1194 | (44.1) |
| Male | 25006 | (55.1) | 11219 | (57.1) | 10570 | (55.1) | 1516 | (55.9) |
| Indeterminate | 77 | (0.2) | 7 | (0.0) | 13 | (0.1) | 0 | (0.0) |
| Missing | 4842 |  | 48 |  | 22 |  | 3 |  |
| **Condition at discharge** |  |  |  |  |  |  |  |  |
| Dead | 7120 | (14.6) | 2929 | (14.9) | 2568 | (13.4) | 549 | (20.3) |
| Alive | 41645 | (85.4) | 16753 | (85.1) | 16611 | (86.6) | 2160 | (79.7) |
| Missing | 1491 |  | 6 |  | 34 |  | 4 |  |
| **Discharge diagnosis / cause of death*** |  |  |  |  |  |  |  |  |
| Congenital malformation | 2,163 | (4.3) | 601 | (3.1) | 1,397 | (7.3) | 219 | (8.1) |
| Prematurity | 13,209 | (26.3) | 8,076 | (41.7) | 5,649 | (29.4) | 678 | (25.0) |
| Infection | 11,445 | (22.8) | 3,132 | (16.2) | 8,025 | (41.8) | 1,520 | (56.1) |
| Intrapartum-related | 17,817 | (35.5) | 1,351 | (7.0) | 5,465 | (28.5) | 913 | (33.7) |
| Jaundice | 2,601 | (5.2) | 933 | (4.8) | 2,454 | (12.8) | 999 | (36.8) |
| Not recorded in medical records | 5,447 | (10.8) | 0 | (0.0) | 154 | (0.8) | 13 | (0.5) |
| Other: reason not specified | 194 | (0.4) | 5,283 | (27.3) | 91 | (0.5) | 110 | (4.1) |
| Other: neonatal reason | 3,584 | (7.1) | 0 | (0.0) | 2,202 | (11.5) | 487 | (18.0) |
| Other: maternal reason | 249 | (0.5) | 0 | (0.0) | 245 | (1.3) | 38 | (1.4) |
| Missing | 21 |  | 318 |  | 9 |  | 2 |  |

**Legend:** *admissions could have more than one diagnosis at discharge

**Abbreviations:** CI, confidence interval; IQR, interquartile range; LBW, low birth weight; VLBW, very low birth weight.

## Additional File 5: *Local ethical approval for the complex evaluation of the implementation of a small and sick newborn care package with NEST360*

| **Country** | **Protocol Title** | **LEC Protocol ID** |
| --- | --- | --- |
| **Kenya** | Using a Health Facility Assessment to Assess Quality of New Born Care in Kenya | MSU/DRPI/MUERC/00810/19 |
| **Malawi** | Using a Health Facility Assessment to Assess Quality of Newborn Care in Malawi | NHSRC 2463 |
| **Nigeria** | Quality Improvement Study of the Implementation of a Package of Trainings and Technologies for the Delivery of Comprehensive Newborn Care in Nigeria: A Multi-Country Study | **LUTH:** ADM/DCST/HREC/APP/3487 |
|  |  | **UCH:** UI/EC/20/0713 |
|  |  | **NHREC:** NHREC/01/01/2007 |
| **Tanzania** | Implementation study to improve the quality of comprehensive newborn care through introduction of the package of Newborn Essential Solutions and Technologies (NEST) in Tanzania | **IHI:**IHI/IRB/01-2021 |
|  |  | **MUHAS:**MUHAS-REC-12-2019-072 |
|  |  | **NIMR:** 3405 |

**Abbreviations**: LEC; Local Ethics Committee, ID; Identity, MSU; Michigan State University, DRPI; Disability Right Promotion International, MUERC; Maseno University Ethics Review Committee, NHSRC; National Health Science Research Committee, LUTH; Lagos University Teaching Hospital, UCH; University College Hospital, NHREC; National Health Research Ethics Committee, IHI; Ifakara Health Institute, MUHAS; Muhimbili University of Health and Allied Science, NIMR; National Institute for Medical Research

**Additional File 6**: ***Blood culture use and antibiotic prescriptions per neonatal unit included in the study***

*Each row represents a neonatal unit.*

| **Country** | **Admissions** | **Infection diagnosis** | **(Row%)** | **Blood culture done** | **(Row%)** | **Received antibiotics** | **(Row%)** | **Blood culture result** | **(Row%)** | **Blood culture positive** | **(Row%)** | **AST done** | **(Row%)** |
| --- | --- | --- | --- | --- | --- | --- | --- | --- | --- | --- | --- | --- | --- |
| Kenya | **1219** | 248 | (20.3) | 0 | (0.0) | 717 | (58.8) | 0 | (0.0) | 0 | (0.0) | 0 | (0.0) |
| Kenya | **1049** | 177 | (16.9) | 0 | (0.0) | 842 | (80.3) | 0 | (0.0) | 0 | (0.0) | 0 | (0.0) |
| Kenya | **3198** | 882 | (27.6) | 0 | (0.0) | 1807 | (56.5) | 0 | (0.0) | 0 | (0.0) | 0 | (0.0) |
| Kenya | **6911** | 729 | (10.5) | 2 | (0.0) | 6476 | (93.7) | 0 | (0.0) | 0 | (0.0) | 0 | (0.0) |
| Kenya | **1786** | 548 | (30.7) | 1 | (0.1) | 1085 | (60.8) | 0 | (0.0) | 0 | (0.0) | 0 | (0.0) |
| Kenya | **1775** | 126 | (7.1) | 4 | (0.2) | 1204 | (67.8) | 0 | (0.0) | 0 | (0.0) | 0 | (0.0) |
| Kenya | **2584** | 247 | (9.6) | 13 | (0.5) | 1365 | (52.8) | 0 | (0.0) | 0 | (0.0) | 0 | (0.0) |
| Kenya | **1278** | 370 | (29.0) | 110 | (8.6) | 662 | (51.8) | 0 | (0.0) | 0 | (0.0) | 0 | (0.0) |
| Kenya | **1977** | 463 | (23.4) | 319 | (16.1) | 1652 | (83.6) | 0 | (0.0) | 0 | (0.0) | 0 | (0.0) |
| Kenya | **5205** | 542 | (10.4) | 1208 | (23.2) | 3456 | (66.4) | 0 | (0.0) | 0 | (0.0) | 0 | (0.0) |
| Malawi | **561** | 150 | (26.7) | 0 | (0.0) | 276 | (49.2) | 0 | (0.0) | 0 | (0.0) | 0 | (0.0) |
| Malawi | **1027** | 158 | (15.4) | 0 | (0.0) | 709 | (69.0) | 0 | (0.0) | 0 | (0.0) | 0 | (0.0) |
| Malawi | **375** | 157 | (41.9) | 0 | (0.0) | 302 | (80.5) | 0 | (0.0) | 0 | (0.0) | 0 | (0.0) |
| Malawi | **706** | 108 | (15.3) | 0 | (0.0) | 177 | (25.1) | 0 | (0.0) | 0 | (0.0) | 0 | (0.0) |
| Malawi | **148** | 40 | (27.0) | 0 | (0.0) | 104 | (70.3) | 0 | (0.0) | 0 | (0.0) | 0 | (0.0) |
| Malawi | **18** | 10 | (55.6) | 0 | (0.0) | 14 | (77.8) | 0 | (0.0) | 0 | (0.0) | 0 | (0.0) |
| Malawi | **1596** | 663 | (41.5) | 0 | (0.0) | 1039 | (65.1) | 0 | (0.0) | 0 | (0.0) | 0 | (0.0) |
| Malawi | **726** | 217 | (29.9) | 0 | (0.0) | 502 | (69.1) | 0 | (0.0) | 0 | (0.0) | 0 | (0.0) |
| Malawi | **1802** | 419 | (23.3) | 0 | (0.0) | 485 | (26.9) | 0 | (0.0) | 0 | (0.0) | 0 | (0.0) |
| Malawi | **1500** | 239 | (15.9) | 0 | (0.0) | 1024 | (68.3) | 0 | (0.0) | 0 | (0.0) | 0 | (0.0) |
| Malawi | **831** | 309 | (37.2) | 0 | (0.0) | 788 | (94.8) | 0 | (0.0) | 0 | (0.0) | 0 | (0.0) |
| Malawi | **859** | 180 | (21.0) | 0 | (0.0) | 487 | (56.7) | 0 | (0.0) | 0 | (0.0) | 0 | (0.0) |
| Malawi | **1170** | 236 | (20.2) | 0 | (0.0) | 450 | (38.5) | 0 | (0.0) | 0 | (0.0) | 0 | (0.0) |
| Malawi | **926** | 241 | (26.0) | 0 | (0.0) | 579 | (62.5) | 0 | (0.0) | 0 | (0.0) | 0 | (0.0) |
| Malawi | **714** | 209 | (29.3) | 0 | (0.0) | 325 | (45.5) | 0 | (0.0) | 0 | (0.0) | 0 | (0.0) |
| Malawi | **1609** | 479 | (29.8) | 0 | (0.0) | 1403 | (87.2) | 0 | (0.0) | 0 | (0.0) | 0 | (0.0) |
| Malawi | **788** | 264 | (33.5) | 0 | (0.0) | 524 | (66.5) | 0 | (0.0) | 0 | (0.0) | 0 | (0.0) |
| Malawi | **1290** | 222 | (17.2) | 0 | (0.0) | 724 | (56.1) | 0 | (0.0) | 0 | (0.0) | 0 | (0.0) |
| Malawi | **1145** | 241 | (21.0) | 0 | (0.0) | 669 | (58.4) | 0 | (0.0) | 0 | (0.0) | 0 | (0.0) |
| Malawi | **1484** | 543 | (36.6) | 0 | (0.0) | 708 | (47.7) | 0 | (0.0) | 0 | (0.0) | 0 | (0.0) |
| Malawi | **1632** | 508 | (31.1) | 1 | (0.1) | 1356 | (83.1) | 1 | (0.1) | 1 | (0.1) | 1 | (0.1) |
| Malawi | **2828** | 491 | (17.4) | 1 | (0.0) | 1098 | (38.8) | 0 | (0.0) | 0 | (0.0) | 0 | (0.0) |
| Malawi | **11745** | 1800 | (15.3) | 6 | (0.1) | 6125 | (52.1) | 5 | (0.0) | 1 | (0.0) | 0 | (0.0) |
| Malawi | **1778** | 341 | (19.2) | 1 | (0.1) | 992 | (55.8) | 0 | (0.0) | 0 | (0.0) | 0 | (0.0) |
| Malawi | **3989** | 1550 | (38.9) | 4 | (0.1) | 2346 | (58.8) | 4 | (0.1) | 0 | (0.0) | 0 | (0.0) |
| Malawi | **2980** | 664 | (22.3) | 3 | (0.1) | 1340 | (45.0) | 2 | (0.1) | 2 | (0.1) | 1 | (0.0) |
| Malawi | **6471** | 2100 | (32.5) | 8 | (0.1) | 3071 | (47.5) | 7 | (0.1) | 0 | (0.0) | 0 | (0.0) |
| Malawi | **1930** | 477 | (24.7) | 3 | (0.2) | 1085 | (56.2) | 1 | (0.1) | 0 | (0.0) | 0 | (0.0) |
| Malawi | **705** | 155 | (22.0) | 2 | (0.3) | 459 | (65.1) | 2 | (0.3) | 0 | (0.0) | 0 | (0.0) |
| Malawi | **1368** | 379 | (27.7) | 4 | (0.3) | 892 | (65.2) | 2 | (0.1) | 0 | (0.0) | 0 | (0.0) |
| Malawi | **2079** | 626 | (30.1) | 8 | (0.4) | 1449 | (69.7) | 0 | (0.0) | 0 | (0.0) | 0 | (0.0) |
| Malawi | **560** | 102 | (18.2) | 3 | (0.5) | 426 | (76.1) | 2 | (0.4) | 0 | (0.0) | 0 | (0.0) |
| Malawi | **371** | 176 | (47.4) | 4 | (1.1) | 306 | (82.5) | 1 | (0.3) | 1 | (0.3) | 1 | (0.3) |
| Malawi | **5776** | 1992 | (34.5) | 88 | (1.5) | 4005 | (69.3) | 21 | (0.4) | 9 | (0.2) | 7 | (0.1) |
| Malawi | **6312** | 1344 | (21.3) | 102 | (1.6) | 4269 | (67.6) | 28 | (0.4) | 9 | (0.1) | 6 | (0.1) |
| Malawi | **287** | 90 | (31.4) | 29 | (10.1) | 144 | (50.2) | 17 | (5.9) | 8 | (2.8) | 6 | (2.1) |
| Malawi | **11078** | 2856 | (25.8) | 2580 | (23.3) | 4870 | (44.0) | 426 | (3.8) | 105 | (0.9) | 28 | (0.3) |
| Nigeria | **601** | 363 | (60.4) | 0 | (0.0) | 601 | (100.0) | 0 | (0.0) | 0 | (0.0) | 0 | (0.0) |
| Nigeria | **504** | 376 | (74.6) | 0 | (0.0) | 504 | (100.0) | 0 | (0.0) | 0 | (0.0) | 0 | (0.0) |
| Nigeria | **174** | 163 | (93.7) | 1 | (0.6) | 170 | (97.7) | 1 | (0.6) | 1 | (0.6) | 1 | (0.6) |
| Nigeria | **45** | 42 | (93.3) | 1 | (2.2) | 45 | (100.0) | 1 | (2.2) | 0 | (0.0) | 0 | (0.0) |
| Nigeria | **210** | 181 | (86.2) | 11 | (5.2) | 198 | (94.3) | 11 | (5.2) | 3 | (1.4) | 3 | (1.4) |
| Nigeria | **1054** | 326 | (30.9) | 309 | (29.3) | 1052 | (99.8) | 116 | (11.0) | 77 | (7.3) | 71 | (6.7) |
| Nigeria | **571** | 477 | (83.5) | 189 | (33.1) | 499 | (87.4) | 180 | (31.5) | 23 | (4.0) | 17 | (3.0) |
| Nigeria | **597** | 291 | (48.7) | 235 | (39.4) | 582 | (97.5) | 233 | (39.0) | 114 | (19.1) | 98 | (16.4) |
| Nigeria | **409** | 378 | (92.4) | 158 | (38.6) | 376 | (91.9) | 153 | (37.4) | 9 | (2.2) | 7 | (1.7) |
| Nigeria | **319** | 135 | (42.3) | 142 | (44.5) | 309 | (96.9) | 142 | (44.5) | 41 | (12.9) | 0 | (0.0) |
| Nigeria | **1109** | 599 | (54.0) | 529 | (47.7) | 1109 | (100.0) | 202 | (18.2) | 96 | (8.7) | 83 | (7.5) |
| Tanzania | **6691** | 3814 | (57.0) | 22 | (0.3) | 6652 | (99.4) | 19 | (0.3) | 1 | (0.0) | 1 | (0.0) |
| Tanzania | **2586** | 2040 | (78.9) | 66 | (2.6) | 2343 | (90.6) | 32 | (1.2) | 17 | (0.7) | 10 | (0.4) |
| Tanzania | **4404** | 1671 | (37.9) | 162 | (3.7) | 4398 | (99.9) | 35 | (0.8) | 23 | (0.5) | 23 | (0.5) |
| Tanzania | **2557** | 547 | (21.4) | 97 | (3.8) | 1308 | (51.2) | 46 | (1.8) | 22 | (0.9) | 19 | (0.7) |
| Tanzania | **7440** | 2283 | (30.7) | 512 | (6.9) | 5693 | (76.5) | 274 | (3.7) | 195 | (2.6) | 167 | (2.2) |
| Tanzania | **4608** | 2978 | (64.6) | 391 | (8.5) | 4219 | (91.6) | 206 | (4.5) | 148 | (3.2) | 147 | (3.2) |
| Tanzania | **2121** | 1352 | (63.7) | 1179 | (55.6) | 1813 | (85.5) | 361 | (17.0) | 194 | (9.1) | 123 | (5.8) |
