# Supplementary material for: Blood culture versus antibiotic use for neonatal inpatients in 61 hospitals implementing with the NEST360 Alliance in Kenya, Malawi, Nigeria, and Tanzania: a cross-sectional study
Source: BMC Pediatr. 2023 Nov 15;23(Suppl 2):568. doi: 10.1186/s12887-023-04343-0 (PMC10652421; doi:10.1186/s12887-023-04343-0)
Supplement: Supplementary file 2 — Additional file 2. STROBE Checklist. Checklist of items that should be included in reports of observational studies. [file 12887_2023_4343_MOESM2_ESM.docx]

**Additional File 2. STROBE checklist**

*Checklist of items that should be included in reports of observational studies.*

|  | Item No. | Recommendation | Included? |
| --- | --- | --- | --- |
| **Title and abstract** | 1 | (*a*) Indicate the study’s design with a commonly used term in the title or the abstract | **Yes** |
|  |  | (*b*) Provide in the abstract an informative and balanced summary of what was done and what was found | **Yes** |
| Introduction | | | |
| Background/rationale | 2 | Explain the scientific background and rationale for the investigation being reported | **Yes** |
| Objectives | 3 | State specific objectives, including any prespecified hypotheses | **Yes** |
| Methods | | | |
| Study design | 4 | Present key elements of study design early in the paper | **Yes** |
| Setting | 5 | Describe the setting, locations, and relevant dates, including periods of recruitment, exposure, follow-up, and data collection | **Yes** |
| Participants | 6 | (*a*) *Cohort study*—Give the eligibility criteria, and the sources and methods of selection of participants. Describe methods of follow-up  *Case-control study*—Give the eligibility criteria, and the sources and methods of case ascertainment and control selection. Give the rationale for the choice of cases and controls  *Cross-sectional study*—Give the eligibility criteria, and the sources and methods of selection of participants | **Yes** |
|  |  | (*b*) *Cohort study*—For matched studies, give matching criteria and number of exposed and unexposed  *Case-control study*—For matched studies, give matching criteria and the number of controls per case | **n/a** |
| Variables | 7 | Clearly define all outcomes, exposures, predictors, potential confounders, and effect modifiers. Give diagnostic criteria, if applicable | **Yes** |
| Data sources/ measurement | 8* | For each variable of interest, give sources of data and details of methods of assessment (measurement). Describe comparability of assessment methods if there is more than one group | **Yes** |
| Bias | 9 | Describe any efforts to address potential sources of bias | **Yes** |
| Study size | 10 | Explain how the study size was arrived at | **Yes** |
| Quantitative variables | 11 | Explain how quantitative variables were handled in the analyses. If applicable, describe which groupings were chosen and why | **Yes** |
| Statistical methods | 12 | (*a*) Describe all statistical methods, including those used to control for confounding | **Yes** |
|  |  | (*b*) Describe any methods used to examine subgroups and interactions | **Yes** |
|  |  | (*c*) Explain how missing data were addressed | **Yes** |
|  |  | (*d*) *Cohort study*—If applicable, explain how loss to follow-up was addressed  *Case-control study*—If applicable, explain how matching of cases and controls was addressed  *Cross-sectional study*—If applicable, describe analytical methods taking account of sampling strategy | **Yes** |
|  |  | (*e*) Describe any sensitivity analyses | **n/a** |

*Continued over page*

| **Results** |  |  |  |
| --- | --- | --- | --- |
| Participants | 13* | (a) Report numbers of individuals at each stage of study—eg numbers potentially eligible, examined for eligibility, confirmed eligible, included in the study, completing follow-up, and analysed | **Yes** |
|  |  | (b) Give reasons for non-participation at each stage | **Yes** |
|  |  | (c) Consider use of a flow diagram | **Yes** |
| Descriptive data | 14* | (a) Give characteristics of study participants (eg demographic, clinical, social) and information on exposures and potential confounders | **Yes** |
|  |  | (b) Indicate number of participants with missing data for each variable of interest | **Yes** |
|  |  | (c) *Cohort study*—Summarise follow-up time (eg, average and total amount) | **Yes** |
| Outcome data | 15* | *Cohort study*—Report numbers of outcome events or summary measures over time | **Yes** |
|  |  | *Case-control study—*Report numbers in each exposure category, or summary measures of exposure | **n/a** |
|  |  | *Cross-sectional study—*Report numbers of outcome events or summary measures | **Yes** |
| Main results | 16 | (*a*) Give unadjusted estimates and, if applicable, confounder-adjusted estimates and their precision (eg, 95% confidence interval). Make clear which confounders were adjusted for and why they were included | **Yes** |
|  |  | (*b*) Report category boundaries when continuous variables were categorised | **Yes** |
|  |  | (*c*) If relevant, consider translating estimates of relative risk into absolute risk for a meaningful time period | **n/a** |
| Other analyses | 17 | Report other analyses done—eg analyses of subgroups and interactions, and sensitivity analyses | **n/a** |
| **Discussion** |  |  |  |
| Key results | 18 | Summarise key results with reference to study objectives | **Yes** |
| Limitations | 19 | Discuss limitations of the study, taking into account sources of potential bias or imprecision. Discuss both direction and magnitude of any potential bias | **Yes** |
| Interpretation | 20 | Give a cautious overall interpretation of results considering objectives, limitations, multiplicity of analyses, results from similar studies, and other relevant evidence | **Yes** |
| Generalisability | 21 | Discuss the generalisability (external validity) of the study results | **Yes** |
| **Other information** |  |  |  |
| Funding | 22 | Give the source of funding and the role of the funders for the present study and, if applicable, for the original study on which the present article is based | **Yes** |

## Abbreviations: n/a; non-applicable
