# Supplementary material for: Blood culture versus antibiotic use for neonatal inpatients in 61 hospitals implementing with the NEST360 Alliance in Kenya, Malawi, Nigeria, and Tanzania: a cross-sectional study
Source: BMC Pediatr. 2023 Nov 15;23(Suppl 2):568. doi: 10.1186/s12887-023-04343-0 (PMC10652421; doi:10.1186/s12887-023-04343-0)
Supplement: Supplementary file 3 — Additional file 3. Variables included in the analysis. Listed in the table are variables by outcome, newborn characteristics, and hospital characteristics. The variable type is also provided, and an explanation as to how variables were transformed and why. [file 12887_2023_4343_MOESM3_ESM.docx]

**Additional File 3: Variables included in the analysis.**

Listed in the table are variables by outcome, newborn characteristics, and hospital characteristics. The variable type is also provided, and an explanation as to how variables were transformed and why.

| **OUTCOME VARIABLES (NID)** | | |
| --- | --- | --- |
| VARIABLE | TYPE | VARIABLE TRANSFORMATION |
| 1. Antibiotic prescribed | Binary | Collected as a binary variable in NID: “yes”; “no”. If “yes”, the antibiotic type was selected from a given list or provided as free text. |
| 2. Blood culture documented | Binary | Collected as a categorical variable in NID: “blood culture done – result unknown”; “blood culture done – result positive”; “blood culture done – result negative”; “blood culture not done”. Recoded into a binary variable as “blood culture done” and “blood culture not done.” |
| **NEWBORN BACKGROUND CHARACTERISTICS (NID)** | | |
| VARIABLE | TYPE | VARIABLE TRANSFORMATION |
| 1. Age at admission (days) | Categorical | Calculated by subtracting date of admission from date of birth and converting the value into a continuous variable with days as units. This categorical variable was then categorised into five age categories: <1, 1-3, 4-6, 7-13 and 14+. All negative intervals were recoded to missing. Most newborn admissions last between 0-3 days which is why this interval was split. |
| 2. Gestational age at admission (weeks) | Categorical | Collected as a continuous variable and categorised into four groups^62^: extremely preterm (less than 28 weeks); very preterm (28 to 32 weeks); moderate to late preterm (32 to 37 weeks), and term (37 weeks or more). |
| 3. Birth weight (g) | Categorical | Collected as a continuous variable and categorised bands to reflect internationally recognised birth weight definitions: very low birth weight <1500g; low birth weight 1500-2499g, normal birth weight 2500-3500g, high birth weight >4000g. |
| 4. Length of admission  (days) | Categorical | Calculated by subtracting date of admission from date of discharge and converting the value into a continuous variable with days as units. This categorical variable was then categorised into six intervals; 1-2 days, 3-13 days, and 14+ days to achieve a reasonable distribution of newborns across groups. All negative intervals were recoded as missing. |
| 5. Sex | Binary | Collected and coded as male, female, or indeterminate. |
| 6. Place of birth | Categorical | Collected as “inborn” or “outborn”. If “outborn”, either” at home”, “in transit”, “at a religious building”, or “another healthcare facility” was specified. Responses were recoded and collapsed into three categories: “born at facility”, “born at another facility”, “born at home”, the latter including “in transit”, and “at a religious building”. |
| 7. Condition at discharge | Binary | Collected and coded as alive or dead. |
| **HOSPITAL CHARACTERISTICS (HFA)** | | |
| VARIABLE | DESCRIPTION | |
| **Laboratory Readiness for Culture** | | |
| Laboratory service available 24/7 | Laboratory is operational 24 hours per day 7 days per week. This does not necessarily mean that the lab is staffed at all times. | |
| Laboratory can do Gram staining | Response options include Available today / Usually available, but not today / Not available, but can be outsourced / Not available today | |
| Laboratory can perform cultures on samples of blood |  |  |
| Laboratory can perform AST on samples of blood |  |  |
| Microscope | Response options include Available today and no stockout in last 4 weeks / Available today, but stockout in last 4 weeks / Not available today | |
| Blood culture bottle (paediatric) |  |  |
| Blood culture bottle (adult) |  |  |
| Petri dishes |  |  |
| Culture media |  |  |
| Sterile picks and loops |  |  |
| Microscope |  |  |
| Protocol for reporting culture results back to neonatal unit | All protocols must be observed by data collector in order to select yes. Response options include Available and easily accessible / Available, but not easily accessible / Not available. (This response is from the perspective of the lab manager/technician). | |
| **Neonatal Unit Readiness for Culture** | | |
| Nurse:newborn ratio (day of HFA visit) | Number of nurses providing exclusive care on the neonatal unit on the day of HFA visit to number of babies in the neonatal unit at the time of the HFA visit | |
| Nurse:newborn ratio (night before HFA visit) | Number of nurses providing exclusive care on the neonatal unit the night before HFA visit to number of babies in the neonatal unit at the time of the HFA visit | |
| Antiseptics | Response options include Available today and no stockout in last 4 weeks / Available today, but stockout in last 4 weeks / Not available today | |
| Gloves |  |  |
| Gauze |  |  |
| Sterile needles or butterfly set |  |  |
| Sterile syringe (any size) |  |  |
| Protocol for early diagnosis and management of neonatal infection | All protocols must be observed by data collector in order to select yes. Response options include Available and easily accessible / Available, but not easily accessible / Not available. (Response from the perspective of the neonatal unit nurse in charge). | |
| Protocol for receiving results from lab & adding to patient files |  |  |
| **General Facility Requirements** | | |
| Connected to central electricity grid | If yes, facility is connected to central electricity grid even if connection to electricity grid is irregular. | |
| Generator cover on neonatal ward | Facility backup power covers the neonatal unit even if not all lighting or equipment are covered by backup power. | |
| Water access | Options include piped water from municipality, borehole (either hand pump or solar pump), other (well (hand pump, electric, or solar), river) | |

**Abbreviations:** NID, neonatal inpatient dataset; HFA, health facility assessment, AST; antimicrobial sensitivity testing.
