# Supplementary material for: Blood culture versus antibiotic use for neonatal inpatients in 61 hospitals implementing with the NEST360 Alliance in Kenya, Malawi, Nigeria, and Tanzania: a cross-sectional study
Source: BMC Pediatr. 2023 Nov 15;23(Suppl 2):568. doi: 10.1186/s12887-023-04343-0 (PMC10652421; doi:10.1186/s12887-023-04343-0)
Supplement: Supplementary file 4 — Additional file 4. Characteristics of eligible newborns admitted to hospitals implementing with NEST360 during the study period, January 2019–August 2022 (N=144,146 newborn records). Table of characteristics of newborn records included in the study, stratified by country. [file 12887_2023_4343_MOESM4_ESM.docx]

**Additional File 4: Characteristics of eligible newborns admitted to hospitals implementing NEST360 during the study period, January 2019-August 2022 (N=144146 newborn records)**

|  | **Malawi** | | **Kenya** | | **Tanzania** | | **Nigeria** | |
| --- | --- | --- | --- | --- | --- | --- | --- | --- |
| **Total Admissions** | **81164** | | **26982** | | **30407** | | **5593** | |
|  | n | (column %) | n | (column %) | n | (column %) | n | (column %) |
| **Age at admission** |  |  |  |  |  |  |  |  |
| <1 day | 59723 | (74) | 21540 | (80) | 19119 | (63) | 2811 | (50) |
| ≥1 to <4 days | 12709 | (16) | 4389 | (16) | 7181 | (24) | 1546 | (28) |
| ≥4 to <7 days | 1887 | (2) | 549 | (2) | 1305 | (4) | 482 | (9) |
| ≥7 to <14 days | 3137 | (4) | 371 | (1) | 1490 | (5) | 387 | (7) |
| ≥14 days | 3708 | (5) | 133 | (1) | 1312 | (4) | 367 | (7) |
| **Gestational age at admission** |  |  |  |  |  |  |  |  |
| Term (≥37 weeks) | 31179 | (45) | 13181 | (54) | 12986 | (50) | 1289 | (37) |
| Late preterm (≥32 to <37 weeks) | 28980 | (41) | 6318 | (26) | 8955 | (35) | 1222 | (35) |
| Very preterm (≥28 to <32 weeks) | 6735 | (10) | 3057 | (12) | 2791 | (11) | 687 | (20) |
| Extremely preterm (<28 weeks) | 3010 | (4) | 1918 | (8) | 1160 | (4) | 297 | (8) |
| *Missing* | *11260* |  | *2508* |  | *4515* |  | *2098* |  |
| **Birth weight** |  |  |  |  |  |  |  |  |
| <1,000g (i.e., ELBW) | 1180 | (1) | 844 | (3) | 654 | (2) | 209 | (4) |
| 1,000-1499g (i.e., VLBW) | 5609 | (7) | 2455 | (9) | 2215 | (7) | 521 | (9) |
| 1,500-2,499g (i.e., LBW) | 23413 | (29) | 7430 | (28) | 9006 | (30) | 1259 | (23) |
| 2,500-3,499g | 36939 | (46) | 11070 | (41) | 13081 | (43) | 1556 | (28) |
| 3,500+g | 14023 | (17) | 5183 | (19) | 5451 | (18) | 2048 | (37) |
| **Length of admission** |  |  |  |  |  |  |  |  |
| <1 day | 5919 | (8) | 1091 | (4) | 1175 | (4) | 252 | (5) |
| ≥1 to <3 days | 30776 | (40) | 7012 | (26) | 9755 | (32) | 714 | (13) |
| ≥3 to <7 days | 27114 | (35) | 9248 | (34) | 11743 | (39) | 1732 | (31) |
| ≥7 to <14 days | 8545 | (11) | 4885 | (18) | 4787 | (16) | 1901 | (34) |
| ≥14 days | 4382 | (6) | 4735 | (18) | 2853 | (9) | 970 | (17) |
| *Missing* | *4428* |  | *11* |  | *94* |  | *24* |  |
| **Sex** |  |  |  |  |  |  |  |  |
| Female | 33460 | (45) | 11563 | (43) | 13750 | (45) | 2462 | (44) |
| Male | 41316 | (55) | 15353 | (57) | 16600 | (55) | 3116 | (56) |
| Indeterminate | 85 | (0) | 10 | (0) | 20 | (0) | 1 | (0) |
| *Missing* | *6,303* |  | *56* |  | *37* |  | *14* |  |
| **Condition at discharge** |  |  |  |  |  |  |  |  |
| Dead | 11375 | (14) | 4032 | (15) | 4117 | (14) | 1047 | (19) |
| Alive | 68103 | (86) | 22730 | (85) | 26236 | (86) | 4538 | (81) |
| *Missing* | *1,686* |  | *220* |  | *54* |  | *8* |  |
| **Discharge diagnosis / cause of death*** |  |  |  |  |  |  |  |  |
| Congenital malformation | 3518 | (4) | 811 | (3) | 2229 | (7) | 406 | (7) |
| Prematurity | 22217 | (27) | 11162 | (42) | 8630 | (28) | 1304 | (23) |
| Infection | 16356 | (20) | 4332 | (16) | 10100 | (33) | 2276 | (41) |
| Intrapartum-related | 25528 | (31) | 1781 | (7) | 6068 | (20) | 843 | (15) |
| Jaundice | 1438 | (2) | 1302 | (5) | 1171 | (4) | 522 | (9) |
| Not recorded in medical records | 7631 | (9) | 0 | (0) | 288 | (1) | 23 | (0) |
| Other: reason not specified | 277 | (0) | 7103 | (27) | 70 | (0) | 61 | (1) |
| Other: neonatal reason | 3819 | (5) | 0 | (0) | 1607 | (5) | 131 | (2) |
| Other: maternal reason | 338 | (0) | 0 | (0) | 225 | (1) | 25 | (0) |
| *Missing* | *42* |  | *491* |  | *19* |  | *2* |  |

**Legend:** *admissions could have more than one diagnosis at discharge

**Abbreviations:** LBW, low birth weight; VLBW, very low birth weight
