# Supplementary material for: Blood culture versus antibiotic use for neonatal inpatients in 61 hospitals implementing with the NEST360 Alliance in Kenya, Malawi, Nigeria, and Tanzania: a cross-sectional study
Source: BMC Pediatr. 2023 Nov 15;23(Suppl 2):568. doi: 10.1186/s12887-023-04343-0 (PMC10652421; doi:10.1186/s12887-023-04343-0)
Supplement: Supplementary file 5 — Additional file 5. Blood culture use and antibiotic prescriptions per neonatal unit included in the study. Table with each row representing a neonatal unit included in the study, reporting total admissions, infections diagnoses, antibiotic prescriptions, and blood cultures done. [file 12887_2023_4343_MOESM5_ESM.docx]

**Additional File 5: Blood culture use and antibiotic prescriptions per neonatal unit included in the study**

*Each row represents a neonatal unit.*

| **Country** | **Admissions** | **Infection diagnosis** | **(Row%)** | **Blood culture**  **done** | **(Row%)** | **Received antibiotics** | **(Row%)** | **Blood culture result** | **(Row%)** | **Blood**  **culture positive** | **(Row%)** | **AST done** | **(Row%)** |
| --- | --- | --- | --- | --- | --- | --- | --- | --- | --- | --- | --- | --- | --- |
| Kenya | **1219** | 248 | (20.3) | 0 | (0.0) | 717 | (58.8) | 0 | (0.0) | 0 | (0.0) | 0 | (0.0) |
| Kenya | **1049** | 177 | (16.9) | 0 | (0.0) | 842 | (80.3) | 0 | (0.0) | 0 | (0.0) | 0 | (0.0) |
| Kenya | **3198** | 882 | (27.6) | 0 | (0.0) | 1807 | (56.5) | 0 | (0.0) | 0 | (0.0) | 0 | (0.0) |
| Kenya | **6911** | 729 | (10.5) | 2 | (0.0) | 6476 | (93.7) | 0 | (0.0) | 0 | (0.0) | 0 | (0.0) |
| Kenya | **1786** | 548 | (30.7) | 1 | (0.1) | 1085 | (60.8) | 0 | (0.0) | 0 | (0.0) | 0 | (0.0) |
| Kenya | **1775** | 126 | (7.1) | 4 | (0.2) | 1204 | (67.8) | 0 | (0.0) | 0 | (0.0) | 0 | (0.0) |
| Kenya | **2584** | 247 | (9.6) | 13 | (0.5) | 1365 | (52.8) | 0 | (0.0) | 0 | (0.0) | 0 | (0.0) |
| Kenya | **1278** | 370 | (29.0) | 110 | (8.6) | 662 | (51.8) | 0 | (0.0) | 0 | (0.0) | 0 | (0.0) |
| Kenya | **1977** | 463 | (23.4) | 319 | (16.1) | 1652 | (83.6) | 0 | (0.0) | 0 | (0.0) | 0 | (0.0) |
| Kenya | **5205** | 542 | (10.4) | 1208 | (23.2) | 3456 | (66.4) | 0 | (0.0) | 0 | (0.0) | 0 | (0.0) |
| Malawi | **561** | 150 | (26.7) | 0 | (0.0) | 276 | (49.2) | 0 | (0.0) | 0 | (0.0) | 0 | (0.0) |
| Malawi | **1027** | 158 | (15.4) | 0 | (0.0) | 709 | (69.0) | 0 | (0.0) | 0 | (0.0) | 0 | (0.0) |
| Malawi | **375** | 157 | (41.9) | 0 | (0.0) | 302 | (80.5) | 0 | (0.0) | 0 | (0.0) | 0 | (0.0) |
| Malawi | **706** | 108 | (15.3) | 0 | (0.0) | 177 | (25.1) | 0 | (0.0) | 0 | (0.0) | 0 | (0.0) |
| Malawi | **148** | 40 | (27.0) | 0 | (0.0) | 104 | (70.3) | 0 | (0.0) | 0 | (0.0) | 0 | (0.0) |
| Malawi | **18** | 10 | (55.6) | 0 | (0.0) | 14 | (77.8) | 0 | (0.0) | 0 | (0.0) | 0 | (0.0) |
| Malawi | **1596** | 663 | (41.5) | 0 | (0.0) | 1039 | (65.1) | 0 | (0.0) | 0 | (0.0) | 0 | (0.0) |
| Malawi | **726** | 217 | (29.9) | 0 | (0.0) | 502 | (69.1) | 0 | (0.0) | 0 | (0.0) | 0 | (0.0) |
| Malawi | **1802** | 419 | (23.3) | 0 | (0.0) | 485 | (26.9) | 0 | (0.0) | 0 | (0.0) | 0 | (0.0) |
| Malawi | **1500** | 239 | (15.9) | 0 | (0.0) | 1024 | (68.3) | 0 | (0.0) | 0 | (0.0) | 0 | (0.0) |
| Malawi | **831** | 309 | (37.2) | 0 | (0.0) | 788 | (94.8) | 0 | (0.0) | 0 | (0.0) | 0 | (0.0) |
| Malawi | **859** | 180 | (21.0) | 0 | (0.0) | 487 | (56.7) | 0 | (0.0) | 0 | (0.0) | 0 | (0.0) |
| Malawi | **1170** | 236 | (20.2) | 0 | (0.0) | 450 | (38.5) | 0 | (0.0) | 0 | (0.0) | 0 | (0.0) |
| Malawi | **926** | 241 | (26.0) | 0 | (0.0) | 579 | (62.5) | 0 | (0.0) | 0 | (0.0) | 0 | (0.0) |
| Malawi | **714** | 209 | (29.3) | 0 | (0.0) | 325 | (45.5) | 0 | (0.0) | 0 | (0.0) | 0 | (0.0) |
| Malawi | **1609** | 479 | (29.8) | 0 | (0.0) | 1403 | (87.2) | 0 | (0.0) | 0 | (0.0) | 0 | (0.0) |
| Malawi | **788** | 264 | (33.5) | 0 | (0.0) | 524 | (66.5) | 0 | (0.0) | 0 | (0.0) | 0 | (0.0) |
| Malawi | **1290** | 222 | (17.2) | 0 | (0.0) | 724 | (56.1) | 0 | (0.0) | 0 | (0.0) | 0 | (0.0) |
| Malawi | **1145** | 241 | (21.0) | 0 | (0.0) | 669 | (58.4) | 0 | (0.0) | 0 | (0.0) | 0 | (0.0) |
| Malawi | **1484** | 543 | (36.6) | 0 | (0.0) | 708 | (47.7) | 0 | (0.0) | 0 | (0.0) | 0 | (0.0) |
| Malawi | **1632** | 508 | (31.1) | 1 | (0.1) | 1356 | (83.1) | 1 | (0.1) | 1 | (0.1) | 1 | (0.1) |
| Malawi | **2828** | 491 | (17.4) | 1 | (0.0) | 1098 | (38.8) | 0 | (0.0) | 0 | (0.0) | 0 | (0.0) |
| Malawi | **11745** | 1800 | (15.3) | 6 | (0.1) | 6125 | (52.1) | 5 | (0.0) | 1 | (0.0) | 0 | (0.0) |
| Malawi | **1778** | 341 | (19.2) | 1 | (0.1) | 992 | (55.8) | 0 | (0.0) | 0 | (0.0) | 0 | (0.0) |
| Malawi | **3989** | 1550 | (38.9) | 4 | (0.1) | 2346 | (58.8) | 4 | (0.1) | 0 | (0.0) | 0 | (0.0) |
| Malawi | **2980** | 664 | (22.3) | 3 | (0.1) | 1340 | (45.0) | 2 | (0.1) | 2 | (0.1) | 1 | (0.0) |
| Malawi | **6471** | 2100 | (32.5) | 8 | (0.1) | 3071 | (47.5) | 7 | (0.1) | 0 | (0.0) | 0 | (0.0) |
| Malawi | **1930** | 477 | (24.7) | 3 | (0.2) | 1085 | (56.2) | 1 | (0.1) | 0 | (0.0) | 0 | (0.0) |
| Malawi | **705** | 155 | (22.0) | 2 | (0.3) | 459 | (65.1) | 2 | (0.3) | 0 | (0.0) | 0 | (0.0) |
| Malawi | **1368** | 379 | (27.7) | 4 | (0.3) | 892 | (65.2) | 2 | (0.1) | 0 | (0.0) | 0 | (0.0) |
| Malawi | **2079** | 626 | (30.1) | 8 | (0.4) | 1449 | (69.7) | 0 | (0.0) | 0 | (0.0) | 0 | (0.0) |
| Malawi | **560** | 102 | (18.2) | 3 | (0.5) | 426 | (76.1) | 2 | (0.4) | 0 | (0.0) | 0 | (0.0) |
| Malawi | **371** | 176 | (47.4) | 4 | (1.1) | 306 | (82.5) | 1 | (0.3) | 1 | (0.3) | 1 | (0.3) |
| Malawi | **5776** | 1992 | (34.5) | 88 | (1.5) | 4005 | (69.3) | 21 | (0.4) | 9 | (0.2) | 7 | (0.1) |
| Malawi | **6312** | 1344 | (21.3) | 102 | (1.6) | 4269 | (67.6) | 28 | (0.4) | 9 | (0.1) | 6 | (0.1) |
| Malawi | **287** | 90 | (31.4) | 29 | (10.1) | 144 | (50.2) | 17 | (5.9) | 8 | (2.8) | 6 | (2.1) |
| Malawi | **11078** | 2856 | (25.8) | 2580 | (23.3) | 4870 | (44.0) | 426 | (3.8) | 105 | (0.9) | 28 | (0.3) |
| Nigeria | **601** | 363 | (60.4) | 0 | (0.0) | 601 | (100.0) | 0 | (0.0) | 0 | (0.0) | 0 | (0.0) |
| Nigeria | **504** | 376 | (74.6) | 0 | (0.0) | 504 | (100.0) | 0 | (0.0) | 0 | (0.0) | 0 | (0.0) |
| Nigeria | **174** | 163 | (93.7) | 1 | (0.6) | 170 | (97.7) | 1 | (0.6) | 1 | (0.6) | 1 | (0.6) |
| Nigeria | **45** | 42 | (93.3) | 1 | (2.2) | 45 | (100.0) | 1 | (2.2) | 0 | (0.0) | 0 | (0.0) |
| Nigeria | **210** | 181 | (86.2) | 11 | (5.2) | 198 | (94.3) | 11 | (5.2) | 3 | (1.4) | 3 | (1.4) |
| Nigeria | **1054** | 326 | (30.9) | 309 | (29.3) | 1052 | (99.8) | 116 | (11.0) | 77 | (7.3) | 71 | (6.7) |
| Nigeria | **571** | 477 | (83.5) | 189 | (33.1) | 499 | (87.4) | 180 | (31.5) | 23 | (4.0) | 17 | (3.0) |
| Nigeria | **597** | 291 | (48.7) | 235 | (39.4) | 582 | (97.5) | 233 | (39.0) | 114 | (19.1) | 98 | (16.4) |
| Nigeria | **409** | 378 | (92.4) | 158 | (38.6) | 376 | (91.9) | 153 | (37.4) | 9 | (2.2) | 7 | (1.7) |
| Nigeria | **319** | 135 | (42.3) | 142 | (44.5) | 309 | (96.9) | 142 | (44.5) | 41 | (12.9) | 0 | (0.0) |
| Nigeria | **1109** | 599 | (54.0) | 529 | (47.7) | 1109 | (100.0) | 202 | (18.2) | 96 | (8.7) | 83 | (7.5) |
| Tanzania | **6691** | 3814 | (57.0) | 22 | (0.3) | 6652 | (99.4) | 19 | (0.3) | 1 | (0.0) | 1 | (0.0) |
| Tanzania | **2586** | 2040 | (78.9) | 66 | (2.6) | 2343 | (90.6) | 32 | (1.2) | 17 | (0.7) | 10 | (0.4) |
| Tanzania | **4404** | 1671 | (37.9) | 162 | (3.7) | 4398 | (99.9) | 35 | (0.8) | 23 | (0.5) | 23 | (0.5) |
| Tanzania | **2557** | 547 | (21.4) | 97 | (3.8) | 1308 | (51.2) | 46 | (1.8) | 22 | (0.9) | 19 | (0.7) |
| Tanzania | **7440** | 2283 | (30.7) | 512 | (6.9) | 5693 | (76.5) | 274 | (3.7) | 195 | (2.6) | 167 | (2.2) |
| Tanzania | **4608** | 2978 | (64.6) | 391 | (8.5) | 4219 | (91.6) | 206 | (4.5) | 148 | (3.2) | 147 | (3.2) |
| Tanzania | **2121** | 1352 | (63.7) | 1179 | (55.6) | 1813 | (85.5) | 361 | (17.0) | 194 | (9.1) | 123 | (5.8) |

**Abbreviations:** AST; antimicrobial sensitivity testing.
