# Supplementary material for: Blood culture versus antibiotic use for neonatal inpatients in 61 hospitals implementing with the NEST360 Alliance in Kenya, Malawi, Nigeria, and Tanzania: a cross-sectional study
Source: BMC Pediatr. 2023 Nov 15;23(Suppl 2):568. doi: 10.1186/s12887-023-04343-0 (PMC10652421; doi:10.1186/s12887-023-04343-0)
Supplement: Supplementary file 6 — Additional file 6. Local ethical approval for the complex evaluation of the implementation of a small and sick newborn care package with NEST360. Table of country protocol titles and Local Ethics Committee protocol identity numbers. [file 12887_2023_4343_MOESM6_ESM.docx]

**Additional File 6: Local ethical approval for the complex evaluation of the implementation of a small and sick newborn care package with NEST360**

| **Country** | **Protocol Title** | **LEC Protocol ID** |
| --- | --- | --- |
| **Kenya** | Using a Health Facility Assessment to Assess Quality of New Born Care in Kenya | MSU/DRPI/MUERC/00810/19 |
| **Malawi** | Using a Health Facility Assessment to Assess Quality of Newborn Care in Malawi | NHSRC 2463 |
| **Nigeria** | Quality Improvement Study of the Implementation of a Package of Trainings and Technologies for the Delivery of Comprehensive Newborn Care in Nigeria: A Multi-Country Study | **LUTH:** ADM/DCST/HREC/APP/3487 |
|  |  | **UCH:** UI/EC/20/0713 |
|  |  | **NHREC:** NHREC/01/01/2007 |
| **Tanzania** | Implementation study to improve the quality of comprehensive newborn care through introduction of the package of Newborn Essential Solutions and Technologies (NEST) in Tanzania | **IHI:**IHI/IRB/01-2021 |
|  |  | **MUHAS:**MUHAS-REC-12-2019-072 |
|  |  | **NIMR:** 3405 |

**Abbreviations**: LEC; Local Ethics Committee, ID; Identity, MSU; Michigan State University, DRPI; Disability Right Promotion International, MUERC; Maseno University Ethics Review Committee, NHSRC; National Health Science Research Committee, LUTH; Lagos University Teaching Hospital, UCH; University College Hospital, NHREC; National Health Research Ethics Committee, IHI; Ifakara Health Institute, MUHAS; Muhimbili University of Health and Allied Science, NIMR; National Institute for Medical Research
